# Reef-associated fishes from the offshore reefs of western Campeche Bank, Mexico, with a discussion of mangroves and seagrass beds as nursery habitats

**DOI:** 10.3897/zookeys.843.33873

**Published:** 2019-05-09

**Authors:** D. Ross Robertson, Omar Domínguez-Dominguez, Yareli Margarita López Aroyo, Rigoberto Moreno Mendoza, Nuno Simões

**Affiliations:** 1 Smithsonian Tropical Research Institute, Balboa, Panamá Smithsonian Tropical Research Institute Balboa Panama; 2 Laboratorio de Biologia Acuatica, Facultad de Biologia, Universidad Michoacana de San Nicolás de Hidalgo, Morelia, Michoacan, Mexico Universidad Michoacana de San Nicolás de Hidalgo Morelia Mexico; 3 Unidad Multidisciplinaria de Docencia e Investigación Sisal (UMDI-SISAL), Facultad de Ciencias, Universidad Nacional Autónoma de México, Puerto de Abrigo s/n, Sisal, CP 97356 Yucatán, Mexico Universidad Nacional Autónoma de México Yucatán Mexico; 4 International Chair for Coastal and Marine Studies, Harte Research Institute for Gulf of Mexico Studies, Texas A and M University – Corpus Christi,Texas, USA Texas A and M University Texas United States of America; 5 Laboratorio Nacional de Resiliencia Costera, Laboratorios Nacionales, CONACYT, Mexico Laboratorio Nacional de Resiliencia Costera, Laboratorios Nacionales Mexico Mexico

**Keywords:** endemic species, invasive species juvenile habitats, reef-fishes, Southwest Gulf of Mexico

## Abstract

A series of small emergent coral reefs and shallow, submerged coralliferous banks are scattered along the western edge of Campeche Bank (southwest Gulf of Mexico), 150–200 km offshore from the Yucatán Peninsula, Mexico. Here a reasonably comprehensive, annotated checklist of reef-associated fishes for one reef, Cayo Arcas (expanded from 162 to 209 species) is presented, with preliminary checklists of such fishes from three other emergent reefs (Cayo Arenas, Triángulo Oeste, Triángulo Este) and four submerged bank reefs (Banco Obispo Norte, Banco Obispo Sur, Banco Nuevo and Banco Pera). During 2017–18 a total of 260 species was observed or collected from those reefs, and previous studies and georeferenced museum records in the global aggregator Fishnet2 added another 101 shallow-living species recorded on or adjacent to those reefs. Some coral-reef fishes are thought to be strongly dependent on seagrass and mangrove areas as nursery habitats for maintenance of their local populations on reefs near to those habitats. The abundance of a number of such “nursery” species on these Campeche reefs indicates otherwise, as there are no seagrass- or mangrove habitats for reef fishes within ~ 150 km of the study reefs. Other isolated Caribbean-area reefs that lack mangroves and, in some cases, seagrasses, also support many such nursery species of reef-fishes.

## Introduction

The southwest Gulf of Mexico has relatively few coral reefs, most of which are quite small. Only a few of these are offshore reefs on the broad, shallow Campeche Bank that extends 200+ km north from the Yucatán Peninsula. This part of the Gulf of Mexico has a different marine environment from the rest of that gulf (Belanger et al. 2012, Lorda et al. 2019). Due in part to the number of endemic reef-fishes found there, the shore-fish fauna of this area forms a discrete biogeographic subunit within one of three major biogeographic subdivisions of the shore-fish fauna of the Greater Caribbean (Robertson and Cramer 2014).

The reef-fish faunas of most of the Campeche Bank offshore reefs have not been documented. Only three of them have substantial published checklists: Alacranes Reef, a large (~ 300 km^2^) emergent reef in the center of the bank and the largest reef in the region (González-Gándara and Arias-González 2001), Madagascar Reef, a tiny (~ 0.25 km^2^), shallow, submerged coralliferous rocky bank ~ 40 km offshore from Sisal, on the northwest coast of the Yucatán peninsula (Zarco-Perelló et al. 2014, Robertson et al. 2016a), and Cayo Arcas, a cluster of three small emergent reefs located near the outer edge of the southwest corner of Campeche Bank (Robertson et al. 2016b).

Here we present an expanded list of non-cryptic and cryptic reef-fishes we observed and collected at Cayo Arcas, and at seven other reefs and submerged banks (Cayo Arenas, Triángulo Oeste, Triángulo Este, Obispo Norte, Obispo Sur, Banco Nuevo and Banco Pera), scattered along the western edge of Campeche Bank, ~ 150 km from the mainland coast during 2017–18. In addition we include a list of fishes that were collected from the vicinity of those reefs and are lodged in the database of the aggregator website Fishnet2 (http://www.fishnet2.net/) by some of the 75 international museums that supply data to that website.

Surveys of local reef-fish faunas serve several purposes. They provide the grist for studies of the biogeography of reef fishes by fleshing out information on the distributions of species. If sites are small and depauperate in habitat diversity their faunal composition provides information that helps identify the importance of different habitats and reef-size for maintaining local populations of different species. Both types of information are useful for indicating the importance of sites for management and conservation purposes. Hence the reef-fish faunas of the reefs we discuss here also are of interest not only because of their location, but also because those reefs are small, isolated and lack two major ancillary habitats that are commonly found adjacent to reefs and used by reef-fishes in other parts of the Greater Caribbean: seagrass beds and mangroves.

## Methods

### Study reefs

**Figure 1.**
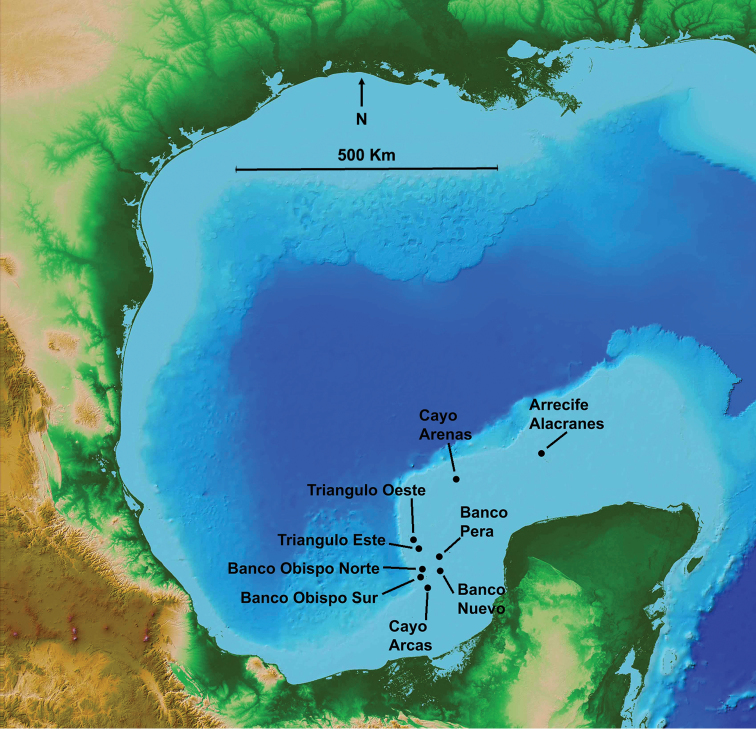
General location of study reefs on Campeche Bank. Base map by NASA.

The set of eight study reefs is spread from the Cayo Arcas complex in the southwest corner of Campeche Bank, to the Cayo Arenas complex, 220 km northeast of Cayo Arcas, at the northwest corner of that Bank (Figure 1). Cayo Arenas (Suppl. material 1: Figures S1–S3) is at 22.11°N, 91.39°W, Triángulo Oeste (Suppl. material 1: Figure S4) at 20.96°N, 92.3°W, Triángulo Este (Suppl. material 1: Figure S5) at 20.91°N, 92.22°W, Banco Obispo Norte (Suppl. material 1: Figure S6) at 20.49°N, 92.20°W, Banco Obispo Sur (Suppl. material 1: Figure S7a,b) at 20.41°N, 92.22°W, Banco Nuevo at 20.55°N, 91.88°W, Banco Pera (Suppl. material 1: Figure S8) at 20.73°N, 91.93°W, and Cayo Arcas (Suppl. material 1: Figures S9, S10) at 20.20°N, 91.97°W. The study reefs include all the emergent reefs on the western edge of Campeche Bank except Cayo Nuevo (21.83°N, 92.09°W), which is located ~ 95 km north of Triángulo Oeste and ~ 78 km southwest of Cayo Arenas, and all the named submerged coralliferous banks except Bancos Ingleses, ~ 15 km east-southeast of Cayo Nuevo. Cayo Arenas and Cayo Arcas each have a manned lighthouse and are permanently staffed by a lighthouse keeper and Mexican Armada marines.

### Reef-fish habitats on the study reefs:

Cayo Arenas, Cayo Arcas and the two Triángulo reefs (Oeste and Este) are emergent reefs or reef complexes, with well-developed coral cover, and coral zonation patterns (Tunnell et al. 2007) similar to those of other reefs on the outer parts of the continental shelf elsewhere in the Greater Caribbean. The reef systems of Cayos Arcas and Arenas each comprise a cluster of three separate emergent reefs spread over an area of ~ 5 km^2^. Triángulo Oeste is a single reef with an emergent area of <0.25 km^2^ , and Triángulo Este (also known as Triángulo Este-Sur) is composed of a 5 km long line of four elongate, narrow (0.5 km wide) emergent reefs separated from each other by shallow-water distances of < 1 km (Suppl. material 1: Fig. S3). Reef development on those reefs extends down to 25–30 m (Logan et al. 1969, Tunnell et al. 2007). The two Obispo banks and Banco Nuevo and Banco Pera are rocky banks that rise to within 10 m (Obispos) and ~ 15 m (Nuevo and Pera) of the surface. While a few small patch reefs on those banks have well developed coral cover, for the most part the surface of the areas of those banks we visited comprised rock, with a thin covering of sand and an abundance of 2–3 m high, tree-like gorgonians, with only small scattered clumps of hard corals 1 m or so in diameter, and numerous large barrel sponges. Logan et al. (1969) described the general geological and morphological characteristics of reefs, submerged coralliferous banks and inter-reef soft bottoms on the Yucatán Peninsular, and Chávez et al. (2007) summarized information about reef morphology and coral zonation patterns of Cayo Arcas. Various aspects of the habitat structure and benthic organisms found on the reefs at Cayo Arcas, Cayo Arenas and the Triángulos reefs also are described and discussed in Tunnell et al. (2007).

### Mangroves and seagrasses on the Campeche Bank reefs

Mangroves and beds of seagrasses (typically *Thalassiatestudinum* Banks ex Koenig, 1805) represent major ancillary habitats adjacent to or within many reefs in the tropical northwest Atlantic. Those two habitats are used by many reef fishes, often as nursery habitats (Nagelkerken et al. 2017). Flores (1992), who is cited as the source of information on mangroves and seagrasses (*T.testudinum*) on Campeche Bank offshore cays by Gulfbase (http://www.gulfbase.org/checklist/pdfs/marine-spp-list.pdf) an aggregator of biological information on the Gulf of Mexico, listed mangroves as being present at Cayo Arcas but not Cayo Arenas. Flores (1992) figure 47 of the distribution of plants on the lighthouse cay at Cayo Arcas shows a few mangrove plants where currently there is a shallow (<1 m deep) pond immediately along the east and southeast side of that cay, inshore from the intertidal reef crest, but he presented no information about their size or abundance. We noticed no mangroves in that area in 2016, but did see several small, young plants in the southeast section of the pond in 2018 (Suppl. material 1: Figure S10). Tunnell and Chapman (2001) surveyed seabird rookeries on Cayo Arenas, Cayo Arcas and Cayo Triángulo Oeste and noted vegetation on those islands, but did not mention mangroves at any of them. We saw mangroves only at Isla Arenas, in 2017. At that site there was a small (~ 30 m diameter) patch of small (up to ~ 1 m high) mangroves with slender (up to several cm diameter) trunks growing on a bottom surfaced entirely with flattened coralline rock chips in the intertidal zone on the southwest side of the Lighthouse island, on the seaward side of a saltwater pond (see Suppl. material 1: Figure S2). At low tides during our stay there (May 2017) those mangroves were completely exposed, with only small (to ~ 25 cm wide), shallow (<2 cm deep) scattered puddles containing water in the mangrove area, a few of which contained a few small gobies (*Bathygobius* sp.), but no other fishes (Suppl. material 1: Figure S3). Hence that mangrove patch does not represent permanently available habitat for reef fishes and would offer little shelter even at high tide. It does not constitute a patch of permanently immersed mangrove nursery habitat of the type commonly used by reef fishes. The situation vis a vis the presence of small stands of stunted, sparsely scattered mangroves on the small, low sandy cays of Campeche Bank on which intertidal habitats are quite likely to change in response to storms may well have changed at Cayo Arenas and Cayo Arcas in 25+ years since Flores’ (1992) research. Tunnell et al. (2007) noted the disappearance of small stands of mangroves growing in small, beach-front ponds next to two sand cays at Alacranes Reef between the 1960s and 2000s. They also noted the presence of a small stand of mangroves growing in a pond at the southeast corner of Perez Island at Alacranes reef. That ~ 0.1 ha stand, much of which was growing on dry land around the pond, was still present in 2016 during the visit by DRR. That pond was separated from the shore by a beach berm, and only intermittently connected to the sea. As with the Cayo Arenas mangrove patch, that on Isla Perez likely does not represent a significant amount of habitat useable as a reef-fish nursery.

There are seven seagrasses in the Gulf of Mexico (Littler and Littler 2000, Green and Short 2003). Three of them, Turtlegrass, *Thalassiatestudinum*; Manatee grass, *Syringodiumfiliforme* Kutzing in Hohenacker, 1860; and Shoal grass, *Halodulewrightii* Ascher, 1868, grow in beds that can be sufficiently dense to provide a shelter-rich habitat useable by reef-associated fishes. No live seagrasses growing on sandy bottoms were seen by us or any other divers from UNAM-Sisal studying shallow water organisms during expeditions to the study reefs in 2016–2018. Those dive sites included lagoonal areas on the leeward sides of emergent reefs and large sand patches on the submerged banks. At Cayo Arcas and Cayo Arenas the cays have shallow (<1m deep) saline ponds bordering the islands that become isolated at low tide; we saw no seagrasses growing in those. We did see substantial amounts of dead seagrass leaves deposited on the beaches of Cayo Arcas in 2016, with much smaller amounts on those beaches in 2018. However, the beaches of Cayo Arenas and the cay at Triángulo Oeste only contained windrows of dead macroalgae. The seagrass windrows on Cayo Arcas were composed of slender, cylindrical, thread-like leaves of *Syringodium* and/or *Halodule*, and no dead blades of *Thalassia* were observed. All those dead seagrass threads were either brown or bleached white, short (mostly <10cm long) and lacked leaf bases with roots attached to them. Similar threads were observed floating in the open sea near Cayo Arcas. Those three species of seagrass are restricted to shallow water in sheltered habitat with fine-sand and mud bottoms: the depth limit of *T.testudinum* is ~ 20m, of *S.filiforme* 25 m, and *H.wrightii* 5 m (Littler and Littler 2000; Short et al. 2010a, b, c). Robertson et al. (2016b) thought that *H.wrightii* might be growing in shallow beach-side ponds of Cayo Arcas. That does not appear to be the case. Thus it is very doubtful that any seagrasses are growing around or very near to the study reefs but missed during our expeditions between 2016 and 2018 as the emergent reefs provide the only sheltered soft substrata in their leeward lagoons, and water around the reefs is deeper than that in which these seagrasses are known to grow.

Turtlegrass was the only seagrass listed by Flores (1992) as present on offshore Campeche reefs, and he indicated it was present at Cayo Arcas, Cayo Arenas, and Cayo Triángulo (i.e., Triángulo Oeste). However, a map (map XIII) of seagrass distribution in the Gulf of Mexico by Green and Short (2003) shows them present inshore along the entire Gulf coast of the Yucatán peninsula and at Alacranes Reef, but nowhere else on Campeche Bank. It seems less likely that seagrasses, which typically grow in sheltered (e.g., lagoonal) habitats, such as that at Alacranes reef, would have vanished from our study reefs. Chávez et al. (2007) noted the absence of seagrasses on “most” Campeche reefs other than Alacranes, which they attributed to a lack of accumulation of fine sediment needed for seagrass establishment. The simplest explanation for the occurrence of the long-dead manatee/shoal grass threads on the beach of Cayo Arcas and floating in water nearby is that they drifted ~ 150 km in from grass beds along the mainland coast. Hence we conclude that two major ancillary habitats, mangroves, and seagrass beds, used by reef fishes throughout much of the wider Caribbean area currently are lacking within ~ 150 km of the offshore reefs of western Campeche Bank.

### Data collection

Cayo Arenas was visited by a group of divers, including DRR, who camped on the island from 22 to 27 May, 2017. During that visit DRR made 14 dives between 0–30 m at different sites on the three reefs that comprise that reef system. During September 8–15, 2017 a group of divers on a live-aboard diving-support vessel, the 30 m long “Barco Isla Mujeres” (see https://www.youtube.com/watch?v=xt-aP0zOFTw.), visited six reefs and banks located between Cayo Arcas and Cayo Arenas. During that expedition DRR, OD-D and RMM made a total of 60 person dives, at Triángulo Oeste (September 8–10, six dives per person between 1–25 m), Triángulo Este (September 11–12; five dives per person between 1–28 m), Banco Obispo Norte (September 13, three dives per person at 9–24m), Banco Obispo Sur (September 14, three dives each at 9–25 m), Banco Nuevo (September 15, two dives each at 18–20m) and Banco Pera (September 15, one dive each at 24 m). During 19–26 June 2018 DRR, RMM and OD-D camped as part of a group of researchers from UNAM-Sisal on Cayo Arcas. Together with two assistants they made a total of 105 person dives at 17 sites on the three reefs that make up that complex. During his dives at the different reefs DRR focused on obtaining a seascape view of the fish fauna, covering large areas and noting the occurrence and abundance of non-cryptic species at different sites. The other divers on the September 2017 and June 2018 trips focused on collecting cryptic fishes using the anesthetic clove oil (10% clove oil in 70% ethanol), and non-cryptic fishes by spearing with multi-pronged pole spears. Collections with clove oil were made in two ways: the anesthetic sprayed into holes without knowing what fishes were inside, and, in other cases, after noticing suitable specimens in holes. Abundance categories given here are the same as those reported in Robertson et al. (2016b) for earlier, similar observations at Cayo Arcas: Rare (1 seen during entire expedition); Uncommon (< 5 total); Occasional (~ 20 total, at multiple dive sites); Common, widespread (scores, at most/all dive sites); Locally common (scores to hundreds at 1–2 dive sites); Abundant, widespread (hundreds to thousands at most/all dive sites).

In addition a search was made on Fishnet2 (www.fishnet2.net; accessed October 5, 2018), for records of fish species collected in two quadrants, one that encompassed Cayo Arenas and adjacent areas on its east and west sides highlighted by Google Earth (quadrat sides: 22.0°N to 22.15°N, 91.05°W to 91.55°W, ~ 885 km^2^), and another that encompassed all the remaining study reefs (quadrat sides: 20.05°N to 21.0°N, 91.5°W to 92.5°W; ~ 11,000 km^2^). We used those quadrats in part to accommodate lack of precision in georeferencing of collection records that might relate to those reefs. We incorporated Fishnet2 records of fish species that could be encountered in shallow reef habitats (< 30 m depth) or occur in pelagic and soft-bottom habitats shallower than 30m adjacent to reefs.

Tissue samples (for genetic analyses) of large, easily recognizable species of reef-fishes and voucher specimens of all small cryptic species that were collected at the study reefs and preserved in ethanol have been deposited in the Ichthyological Collection of the Laboratorio de Biología Acuática, Universidad Michoacana de San Nicolás de Hidalgo, México (SEMARNAT registration number MICH-PEC-227-07-09).

## Results

Table 1 includes a list of 364 fish species from 75 families now known from the west Campeche offshore reefs. Of those 360 are bony fishes, which include 260 species that we observed or collected on the study reefs between 2016–2018. In addition there are nine other species that were observed or collected on those reefs by Chávez (1966) and by Garduño (1988) but not seen by us, and museum records of 136 species in the two quadrats, 96 of which were not recorded by us on the reefs. The 136 include 48 soft-bottom species (46 of which we did not record on the reefs), 31 pelagic species (20 not recorded by us), and 57 species that occur on hard reef substrata (as well as other substrata in some cases), 30 of them not recorded by us. Robertson et al. (2016b) reported a fauna of 162 species from Cayo Arcas; our 2016–18 sampling increased that to 209 species, primarily as a result of collection of 35 cryptic species using clove-oil anesthetic.

**Table 1.** List of fishes observed and collected on 8 reefs on the western edge of Campeche Bank, and of museum records of species of shallow water fishes collected in two quadrats that incorporate those reefs.

Taxon Name	Species Habitat	Triángulo Oeste	Triángulo Este	Obispo Norte	Obispo Sur	Banco Nuevo	Banco Pera	Arenas	Arcas 2016–18	No. reefs	Arenas quadrat	Arcas quadrat
**GINGLYMOSTOMATIDAE**												
*Ginglymostomacirratum*	R&SB	R	R	R				R		4		P
**CARCHARHINIDAE**												
*Carcharhinusbrevipinna*	P		R	R						2		
*Carcharhinuslimbatus* p	P							**C^66^**		1		
*Carcharhinusperezii*	P							R		1		
**SPHYRNIDAE**												
*Sphyrnalewini*	P							R		1		
**RAJIDAE**												
*Rostrorajatexana*	SB											P
**UROTRYGONIDAE**												
*Urobatisjamaicensis* * p	SB				R				R	2		
**DASYATIDAE**												
*Hypanusamericanus* p	SB			R	R				U	3		
**AETOBATIDAE**												
*Aetobatusnarinari* p	SB							R	P	2		
**MEGALOPIDAE**												
*Megalopsatlanticus* p	P	U						U	LC	3		
**ALBULIDAE**												
*Albulavulpes*	SB											P
**MURAENIDAE**												
*Echidnacatenata* *	C,R								P^2018^	1		
*Enchelycorecarychroa* *	C,R	P							P^2018^	2		
*Enchelycorenigricans* p	C,R	P				P		**C^66^**		3		
*Gymnothoraxfunebris* * p	SC,R	**C^66^**						P **G^88^**		2		
*Gymnothoraxmiliaris* *	C,R	P	P					P		3		
*Gymnothoraxmoringa* p	SC,R	**C^66^**	P	P				**C^66^**	P	5		
*Gymnothoraxnigromarginatus*	SB											P
*Gymnothoraxvicinus*	C,R				P					1		
**OPHICHTHIDAE**												
*Ahliaegmontis*	SB,C										P	P
*Myrophisplatyrhynchus*	SB,C											P
**ENGRAULIDAE**												
*Anchoviellaperfasciata*	P											P
*Engrauliseurystole*	P											P
**CLUPEIDAE**												
*Etrumeusacuminatus*	P											P
*Jenkinsialamprotaenia* p	P							A	A	2		P
*Opisthonemaoglinum* *	P								P^2018^	1		P
*Sardinellaaurita*	P											P
**SYNODONTIDAE**												
*Synodusfoetens*	SB											P
*Synodusintermedius*	SB,R		R					R		2		P
*Synoduspoeyi*	SB											P
*Synodussynodus* *	SB,R								R^2018^	1		
*Trachinocephalusmyops*	SB											P
**OPHIDIIDAE**												
*Brotulabarbata*	C											P
*Lepophidiumbrevibarbe*	SB,C											P
**DINEMATICHTHYIDAE**												
*Ogilbiacayorum* * p	R,C	P	P	P	P			**C^66^**		5		
**BATRACHOIDIDAE**												
*Porichthysplectrodon*	SB											P
**ANTENNARIIDAE**												
*Antennariusscaber*	C,R											P
**OGCOCEPHALIDAE**												
*Halieutichthysaculeatus*	SB											P
*Halieutichthysbispinosus*	SB											P
*Ogcocephaluscorniger*	SB											P
*Ogcocephaluscubifrons*	SB											P
*Ogcocephalusparvus*	SB											P
**ATHERINIDAE**												
*Atherinaharringtonensis* * p	P		LC						A	2		
*Atherinomorusstipes* p	P	**C^66^**						LA	A	3		P
**EXOCOETIDAE**												
*Cheilopogoncyanopterus*	P											P
*Cheilopogonheterurus*	P											P
*Parexocoetusbrachypterusbrachypterus*	P											P
*Parexocoetushillianus* *	P			P	P					2		P
**HEMIRAMPHIDAE**												
*Euleptorhamphusvelox*	P											P
*Hemiramphusbrasiliensis*	P											P
*Hyporhamphusunifasciatus*	P											P
**BELONIDAE**												
*Ablenneshians*	P											P
*Platybeloneargalusargalus* *	P		P							1		P
*Tylosurusacusacus*	P											P
*Strongyluranotata* p	P							**G^88^**		1		
**HOLOCENTRIDAE**												
*Neoniphonmarianus* p	R,SC	R						UC **G^88^**		2		
*Holocentrusadscensionis* p	R			O			P	C	C	4		P
*Holocentrusrufus* * p	R	O	LC	O	0	P	P	C **G^88^**	O	8		P
*Myripristisjacobus* * p	R							O	U	2		
*Neoniphoncoruscum*	R,SC					P				1		
*Neoniphonvexillarium* * p	R,SC	**C^66^**				P		C	U	4		
*Plectrypopsretrospinis* p	R,C							**C^66^**		1		
**SYNGNATHIDAE**												
*Halicampuscrinitus* *	R,C								P^2018^	1		
**AULOSTOMIDAE**												
*Aulostomusmaculatus* * p	R	O	O	O	O	P	P	O **G^88^**	U	8		
**FISTULARIIDAE**												
*Fistulariatabacaria*	R,SB											P
**DACTYLOPTERIDAE**												
*Dactylopterusvolitans*	SB											P
**SCORPAENIDAE**												
*Pteroisvolitans* * p	R	R	UC		U			U	O	5		
*Scorpaenabrasiliensis*	R,SC											P
*Scorpaenacalcarata*	R,SC											P
*Scorpaenadispar*	R,C											P
*Scorpaenaplumieri* p	R,SC	**C^66^**								1		P
*Scorpaenodescaribbaeus* *	R,C								P^2018^	1		
*Scorpaenodestredecimspinosus* *	R,C								P^2018^	1		
**TRIGLIDAE**												
*Prionotusophryas*	SB											P
*Prionotuspunctatus*	SB											P
*Prionotusroseus*	SB											P
*Prionotusrubio*	SB											P
*Prionotusstearnsi*	SB										P	P
**EPINEPHELIDAE**												
*Cephalopholiscruentata* * p	R	C	C	C	C	C		C **G^88^**	C	7		
*Cephalopholisfulva* p	R		R					**G^88^**	R	3		P
*Cephalopholisfurcifer* * p	P	C	U					LC	C	4		P
*Epinephelusadscensionis* * p	R	R	O	O	O	U	P	O	C	8		P
*Epinephelusguttatus* * p	R	U	O	O	O	R	P	O	C	8		P
*Epinephelusitajara* p	R							**C^66^**		1		
*Epinephelusmorio* *	R,SB			1						1		
*Epinephelusstriatus* p	R	R						**C^66^ G^88^**		2		
*Hyporthodusflavolimbatus*	R,SB										P	
*Hyporthodusniveatus*	R,SB											P
*Mycteropercaacutirostris* *	R			R						1		
*Mycteropercabonaci* p	R		R					**C^66^**	C	3		P
*Mycteropercainterstitialis* p	R	O	O	O	U	0		O	LC	7		
*Mycteropercamicrolepis* p	R								U	1		
*Mycteropercaphenax* p	R								U	1		P
*Mycteropercatigris* p	R	**C^66^**						**C^66^**	C	3		
*Mycteropercavenenosa*	R	R	U		U			O	R^2018^	5		
**SERRANIDAE**												
*Centropristisocyurus*	SB											P
*Diplectrumbivittatum* p	SB							R		1		P
*Diplectrumformosum*	SB											P
*Hypoplectrusaberrans* * p	R	R	O	U	U		P	U	LC	7		
*Hypoplectrusecosur* * p	R		R	R		O	0		LC	5		
*Hypoplectrusfloridae* *	R								R^2018^	1		
*Hypoplectrusgemma* * p	R	O	O	U		U		U	R	6		
*Hypoplectrusguttavarius* p	R							**C^66^**		1		
*Hypoplectrusindigo* * p	R	R	R						U	3		
*Hypoplectrusmaculiferus* p	R		R						R	2		
*Hypoplectrusnigricans* * p	R		R	R		R			0	4		
*Hypoplectruspuella* * p	R	O	O	O		O	P	C	O	7		
*Hypoplectrusrandallorum* * p	R	R	U	R					R	4		
*Hypoplectrusunicolor* * p	R	R	O					R	R	4		
*Pseudogrammagregoryi* *	C,R			P					P^2018^	2		
*Rypticusbistrispinus*	C											P
*Serranusbaldwini* * p	R			U	O				R	3		
*Serranustabacarius* * p	R			LC	O			U	LC	4		
*Serranustigrinus* * p	R	O	O	O	O			C	C	6		
*Serranustortugarum* * p	R	LC	LC		O	0		LC	LC	6	P	
**GRAMMATIDAE**												
*Grammaloreto* * p	R	C	C	C	C		R	LC	C	7		
**OPISTOGNATHIDAE**												
*Lonchopisthusmicrognathus*	SB,C											P
*Opistognathusaurifrons* * p	SB		LC	R	O	O	P	LC	LC	7		
*Opistognathusmacrognathus* *	SB,C								P^2018^	1		
**PRIACANTHIDAE**												
*Heteropriacanthuscruentatus* p	R,SC							R		1	P	
*Priacanthusarenatus*	R											P
**APOGONIDAE**												
*Apogonaurolineatus* p	C,R							P		1		
*Apogonbinotatus* *	C,R	P	P	P	P	P			P^2018^	6	P	
*Apogonlachneri* *	C,R	P								1		
*Apogonmaculatus* *	C,R	P	P	P	P	P		P	P^2018^	7		
*Apogonplanifrons* *	C,R		P							1		
*Apogontownsendi*	C,R							P		1		
*Astrapogonpuncticulatus*	C,R										P	
*Astrapogonstellatus* *	C,R			P		P				2		
*Phaeoptyxconklini* *	C,R	P	P	P					P^2018^	4		
*Phaeoptyxpigmentaria* *	C,R	P	P	P		P			P^2018^	5		
*Phaeoptyxxenus* *	C,R	P	P							2		
**MALACANTHIDAE**												
*Malacanthusplumieri* * p	SB	U	R		U			LC	R	5		
**CORYPHAENIDAE**												
*Coryphaenahippurus*	P											P
**RACHYCENTRIDAE**												
*Rachycentroncanadum* p	P								U	1		
**ECHENEIDAE**												
*Echeneisnaucrates*	P							R		1		P
**CARANGIDAE**												
*Caranxbartholomaei* p	BP	U							U	2		
*Caranxcrysos* * p	BP					O	C		C	3		P
*Caranxhippos* p	BP							**C^66^**		1		
*Caranxlatus* p	BP	R	O		O			U **G^88^**	C	5		P
*Caranxlugubris* p	BP	U						O **G^88^**	U	3		
*Caranxruber* p	BP	O	C	O	C	UC		LA **G^88^**	LC	7		
*Chloroscombruschrysurus*	BP											P
*Decapterusmacarellus* p	P								LC	1		
*Decapteruspunctatus*	P											P
*Elagatisbipinnulata*	P								R^2018^	1		
*Selarcrumenophthalmus*	P											P
*Selenesetapinnis*	BP											P
*Selenevomer*	BP								R^2018^	1		P
*Seriolarivoliana*	BP					O			R^2018^	2		P
*Trachinotuscarolinus* p	BP								R	1		
*Trachuruslathami*	P											P
**LUTJANIDAE**												
*Lutjanusanalis* p	R						R	**G^88^**	O	3		
*Lutjanusapodus*	R		U					C	U^2018^	3		
*Lutjanusbuccanella*	R				R			U	O	3		P
*Lutjanuscampechanus* p	R										P	P
*Lutjanuscyanopterus*	R							R	R^2018^	2		
*Lutjanusgriseus* * p	R	C	C	O	U	C	U	C **G^88^**	C	8		
*Lutjanusjocu* * p	R	O	C	U		U			C	5		
*Lutjanusmahogoni* p	R							C	O	2		
*Lutjanussynagris* p	R							O	O	2		P
*Ocyuruschrysurus* * p	BP	C	A	A	C	C	C	A **G^88^**	A	8		
*Rhomboplitesaurorubens*	R,SB											P
**GERREIDAE**												
*Diapterusrhombeus*	SB											P
*Eucinostomusgula* p	SB							O	LC	2		P
*Gerrescinereus* p	SB							O	U	2		
**HAEMULIDAE**												
*Anisotremussurinamensis* *	R								LC ^2018^	1		
*Anisotremusvirginicus* p	R			R		R		**G^88^**	LC	4		
*Brachygenyschrysargyreum* * p	R		O						LC	2		
*Emmelichthyopsatlanticus* p	R	A	LA	C	C	U			LC	6		
*Haemulonaurolineatum* * p	R							LC **G^88^**	C	2		P
*Haemuloncarbonarium* p	R		U	U	O			R	U	5		
*Haemulonflavolineatum* * p	R	C	C	C	C	C		C **G^88^**	C	7		
*Haemulonmacrostomum* p	R	R	O						U	3		
*Haemulonmelanurum* p	R		O					U	U	3		
*Haemulonplumierii* * p	R		O	O	O	O	C		U	6		
*Haemulonsciurus* p	R							R	U	2		
*Haemulonstriatum* p	R								LC	1		P
*Haemulonvittatum* * p	R	A	LA	C				C	LC	5		
**SPARIDAE**												
*Calamuscalamus* p	SB		R		R	U	U	O	C	6		
*Calamusnodosus* p	SB	R		R				O	C	4		
*Lagodonrhomboides*	SB											P
*Stenotomuscaprinus*	SB											P
**POLYNEMIDAE**												
*Polydactylusoctonemus*	SB											P
**SCIAENIDAE**												
*Cynoscionarenarius*	SB											P
*Cynoscionnothus*	SB											P
*Equetuslanceolatus*	R											P
*Equetuspunctatus* p	R	R	R	R				R	U	5		
*Micropogoniasundulatus*	SB											P
*Odontosciondentex*	R		O							1		
*Parequesacuminatus* p	R					O	U	**G^88^**	U	4		P
*Parequesumbrosus*	R											P
**MULLIDAE**												
*Mulloidichthysmartinicus* * p	R,SB	C	C	O	C	U		C **G^88^**	C	7		
*Mullusauratus*	SB											P
*Pseudupeneusmaculatus* * p	R,SB	R	U	O	O	O	O	O **G^88^**	LC	8		
*Upeneusparvus*	SB											P
**PEMPHERIDAE**												
*Pempherisschomburgkii* * p	R	U	U					LC **G^88^**	LC	4		
**KYPHOSIDAE**												
*Kyphosusbigibbus* p	R	U						R	O	3		
*Kyphosuscinerascens* p	R	U						U	C	3		
*Kyphosussectatrix* p	R	C	C					C **G^88^**	C	4		
*Kyphosusvaigiensis* p	R	LC	LC					C	C	4		
**CHAETODONTIDAE**												
*Chaetodoncapistratus* * p	R	O		U		U		O	O	5		
*Chaetodonocellatus* * p	R	O	O	O	O	U		C **G^88^**	C	7		P
*Chaetodonsedentarius* * p	R	O	O	O	O	O	P	C **G^88^**	C	8		
*Chaetodonstriatus* * p	R							U **G^88^**	R^2018^	2		
*Prognathodesaculeatus* p	R							R		1		
**POMACANTHIDAE**												
*Holacanthusbermudensis* p	R	U	O			O	P	U **G^88^**	U	6		
*Holacanthusciliaris* * p	R		U	U	U			U **G^88^**	C	5		
*Holacanthustricolor* * p	R							R **G^88^**	R	2		
*Pomacanthusarcuatus* * p	R	U	U	U	U	R	P	O **G^88^**	LC	8		
*Pomacanthusparu* * p	R	O	U			R	P	O **G^88^**	LC	6		P
**CIRRHITIDAE**												
*Amblycirrhituspinos* p	R,C							P	R	2		
**POMACENTRIDAE**												
*Abudefdufsaxatilis* * p	R	C	A	C	C			A **G^88^**	A	6		
*Abudefduftaurus*	R								P^2018^	1		
*Chromiscyanea* * p	R	C	C	O	O			LC **G^88^**	LC	6		
*Chromisenchrysura* *	R								R^2018^	1		
*Chromisinsolata* p	R							R **G^88^**	LC	2		
*Chromismultilineata* *	R	A	A	A	A	LC	LC	A **G^88^**	A	8		
*Chromisscotti* * p	R	C	A	C	C	C	C	LC	C	8		
*Microspathodonchrysurus* * p	R	C	C	O	O			C **G^88^**	C	6		
*Neopomacentruscyanomos* * p	R	C	A	O	C	A	C	LC	A	8		
*Stegastesadustus* * p	R	A	C	C	C			A **G^88^**	A	6		
*Stegastesdiencaeus* * p	R	U						U	R	4		
*Stegastesleucostictus* * p	R	LC	LC	U				LC **G^88^**	LC	5		
*Stegastespartitus* * p	R	A	A	A	A	U		A **G^88^**	A	7		
*Stegastesplanifrons* * p	R	C	C	C	C	C		O **G^88^**	A	7		
*Stegastesxanthurus* * p	R	A	C	C	C	C	C	C **G^88^**	A	8		
**LABRIDAE**												
**Labrinae**												
*Bodianuspulchellus* p	R		R						LC	2		P
*Bodianusrufus* * p	R	O	O	O	O	U		C **G^88^**	C	7		
*Clepticusparrae* * p	R	A	A	A	C	C		A **G^88^**	LC	7		
*Halichoeresbivittatus* p	R	C	C	C	C	C	C	A **G^88^**	A	8		
*Halichoeresburekae* * p	R	A	A	A	A	A	A	A	A	8		
*Halichoeresgarnoti* * p	R	C	C	C	C	C	C	C **G^88^**	C	8		
*Halichoeresmaculipinna* *	R	C	C	C	C	C	C	A **G^88^**	C	8		
*Halichoerespictus*	R											P
*Halichoerespoeyi* p	R								R	1		
*Halichoeresradiatus* * p	R	O	O	O	O	O		**G^88^**	C	7		
*Lachnolaimusmaximus* * p	R,SB		R		U	U	R	**G^88^**	U	6		
*Thalassomabifasciatum* * p	R	C	C	C	C	C	C	A **G^88^**	A	8	P	
*Xyrichtysmartinicensis*	SB,R					U		U		2		
*Xyrichtysnovacula*	SB							U	U^2018^	2		
*Xyrichtyssplendens* * p	SB					O		U	U	3		
**Scarinae**												
*Cryptotomusroseus* * p	R	R		O	O	O	U	U	O	7		
*Nicholsinausta*	R											P
*Scaruscoelestinus* p	R		U					U **G^88^**	O	3		
*Scaruscoeruleus* * p	R		R				O	U **G^88^**	C	4		
*Scarusguacamaia* p	R								O	1		
*Scarusiseri* * p	R	C	C	C	C	C	C	C **G^88^**	C	8		
*Scarustaeniopterus* * p	R	U						C **G^88^**	C	3		
*Scarusvetula* * p	R	C	C	C	C	O		A **G^88^**	C	7		
*Sparisomaatomarium* * p	R	R	U	O	O	O		U	LC	7		
*Sparisomaaurofrenatum* * p	R	C	C	C	C	C	C	LC **G^88^**	C	8		
*Sparisomachrysopterum* p	R	R	O		U	O	O	A **G^88^**	O	7		
*Sparisomaradians* * p	R		O	O	O	U	O	U	C	7		
*Sparisomarubripinne* p	R	O	O	R	U			LC **G^88^**	LC	6		
*Sparisomaviride* * p	R	C	C	C	C	O		C	C	7		
**URANOSCOPIDAE**												
*Astroscopusy-graecum*	SB,C		P							1		
**TRIPTERYGIIDAE**												
*Enneanectesaltivelis* *	R,SC		P	P	P		P		P^2018^	5		
*Enneanectesboehlkei* * p	R,SC			P				P	P	3		
*Enneanectesjordani* *	R,SC	**C^66^**	P	P					P^2018^	4		
**DACTYLOSCOPIDAE**												
*Gillellusgreyae* *	SB,C								P^2018^	1		
*Gillellusuranidea* *	SB,C			P					P^2018^	2		
**BLENNIIDAE**												
*Entomacrodusnigricans* * p	R,SC	P						**C^66^**	U	3		
*Hypleurochilusbermudensis* *	R,SC	P	P	P						3		
*Hypsoblenniusinvemar* * p	R,SC								LC	1		
*Ophioblenniusmacclurei* * p	R,SC	O		O	O			LC **G^88^**	C	5		
*Parablenniusmarmoreus* * p	R,SC							P	LC	2		
*Scartellacristata* * p	R,SC	**C^66^**							LC	2		
**LABRISOMIDAE**												
*Gobioclinusbucciferus* p	R,C	**C^66^**								1		
*Gobioclinusgobio* *	R,C	P		P					P^2018^	3		
*Gobioclinusguppyi* * p	R,C	P	P		P	P		**C^66^**	P^2018^	6		
*Gobioclinushaitiensis* *	R,C		P		P				P^2018^	3		
*Gobioclinuskalisherae* * p	R,C	P	P	P	P			**C^66^**	P^2018^	6		
*Labrisomusnuchipinnis* *	R,C	**C^66^**							P^2018^	2		
*Malacoctenusaurolineatus* * p	R,SC	**C^66^**						LC	LC	3		
*Malacoctenusmacropus* * p	R,SC	O	U				O	C	C	5		
*Malacoctenustriangulatus* * p	R,SC	O	O	O	O	O	O	C	C	8		
*Paraclinusfasciatus* *	R,C								P^2018^	1		
*Paraclinusmarmoratus* *	R,C					P	P			2		
*Paraclinusnigripinnis* *	R,C				P					1		
*Starksiaocellata* * p	R,C	P	P	P	P			P	P	6		
**CHAENOPSIDAE**												
*Acanthemblemariaaspera* *	R,SC	P	P	P	P	P	P	P	P^2018^	8		
*Emblemariapandionis* *	SB,C	P			P				P^2018^	3		
*Emblemariopsisdiaphana* *	R,C		P		P		P	P	P^2018^	5		
*Stathmonotushemphillii* *	R,C								P^2018^	1		
**GOBIESOCIDAE**												
*Gobiesoxstrumosus*	R,C											P
**GOBIIDAE**												
*Barbuliferceuthoecus* *	R,C								P^2018^	1		
*Bathygobiusantilliensis* * p, 1	R,SC	**C^66^**							P^2018^	1		
*Coryphopterusalloides* *	R,C		P							1		
*Coryphopterusdicrus* * p	R,SB,SC	O	O	O	O	O	P	LC	A	8		
*Coryphopteruseidolon* *	R,SC	P	P	P		P		U	P^2018^	6		
*Coryphopterusglaucofraenum* p	SB,SC	P	P				P	C	A	5		
*Coryphopterushyalinus* * p	R	A	A	P	P	P	P	A	A	8		
*Coryphopteruslipernes* *	R,SC			P						1		
*Coryphopteruspersonatus* * p	R	A	A	P	P	P	P	A	A	8		
*Coryphopterustortugae* *	R,SC	P		P					P^2018^	3		
*Ctenogobiussaepepallens* *	SB,SC								P^2018^	1		
*Ctenogobiusstigmaturus* p	SB,SC	**C^66^**								1		
*Elacatinusoceanops* *	R,SC	C	C	O	C	O	O	O	C	8		
*Elacatinusxanthiprora* p	R,C								UC	1		
*Gnatholepisthompsoni* * p	SB,SC	C	C	C	C	O	O	A	A	8		
*Gobiosomagrosvenori*	R,C											P
*Gobulusmyersi* *	R,C								P^2018^	1		
*Lythrypnusminimus* *	R,C			P					P^2018^	2		
*Lythrypnusnesiotes* *	R,C	P	P	P	P					4		
*Lythrypnusspilus* *	R,C								P^2018^	1		
*Priolepishipoliti* *	R,C			P					P^2018^	2		
*Ptereleotriscalliura* p	SB		U		U			U	LC	4		
*Ptereleotrishelenae* *	SB		P							1		
*Risorruber* *	R,C			P						1	P	
**ACANTHURIDAE**												
*Acanthuruschirurgus* p	R	O	U	O	O			C **G^88^**	C	6		
*Acanthuruscoeruleus* * p	R	C	C	U	O			A **G^88^**	C	6		
*Acanthurustractus* * p	R	C	O	U	O	U		A **G^88^**	C	7		
**SPHYRAENIDAE**												
*Sphyraenabarracuda* * p	P	C	O	O	U	U	R	A **G^88^**	O	8		
*Sphyraenaborealis*	P			A					LC^2018^	2		P
*Sphyraenaguachancho*	P											P
**SCOMBRIDAE**												
*Scomberjaponicus*	P											P
*Scomberomoruscavalla*	P											P
**PARALICHTHYIDAE**												
*Citharichthysmacrops*	SB,C											P
*Cyclopsettachittendeni*	SB,C											P
*Cyclopsettafimbriata*	SB,C											P
*Etropuscrossotus*	SB,C											P
*Syaciumgunteri*	SB,C											P
*Syaciumpapillosum*	SB,C											P
**BOTHIDAE**												
*Bothuslunatus* *	R,SB								R^2018^	1		
*Bothusocellatus* *	SB,C								P^2018^	1		
*Bothusrobinsi*	SB,C											P
**ACHIRIDAE**												
*Gymnachirusnudus*	SB,C											P
*Gymnachirustexae*	SB,C											P
**CYNOGLOSSIDAE**												
*Symphuruscivitatium*	SB,C											P
*Symphurusdiomedeanus*	SB,C											P
*Symphurusminor*	SB,C										P	
*Symphurusoculellus*	SB,C											P
*Symphurusplagiusa*	SB,C											P
*Symphurusurospilus*	SB,C											P
**BALISTIDAE**												
*Balistescapriscus* p	R								R	1		P
*Balistesvetula* p	R							U **G^88^**	R	2		
*Canthidermissufflamen* * p	R	C	O	U	U		R	U **G^88^**	O	7		
*Melichthysniger* p	R	C	O	O	U	U		LC **G^88^**	LC	7		
*Xanthichthysringens* p	R							**G^88^**	R	2		
**MONACANTHIDAE**												
*Aluterusheudelotii*	R											P
*Aluterusscriptus* p	R	R		R				LC **G^88^**	O	4		
*Cantherhinesmacrocerus* p	R		R		U			U		3		
*Cantherhinespullus* * p	R			O				O **G^88^**	R	3		
*Monacanthusciliatus*	R										P	P
*Monacanthustuckeri* *	R				O	P			LC	3	P	
*Stephanolepishispidus*	R											P
**OSTRACIIDAE**												
*Acanthostracionpolygonius* p	R							R		1		
*Acanthostracionquadricornis*	R											P
*Lactophrysbicaudalis* *	R								R	1		
*Lactophrystriqueter* * p	R	O	O	O	O	R	R	O **G^88^**	O	8		P
**TETRAODONTIDAE**												
*Canthigasterjamestyleri*	R		P							1		
*Canthigasterrostrata* * p	R	C	C	C	C	O	O	C **G^88^**	C	8		
*Lagocephaluslaevigatus*	SB											P
*Sphoeroidesdorsalis*	R											P
*Sphoeroidespachygaster*	R											P
*Sphoeroidesspengleri* *	R								R^2018^	1		P
*Sphoeroidestestudineus* p	R								R	1		
**DIODONTIDAE**												
*Diodonholocanthus*	R	R						R		2		
*Diodonhystrix* p	R	R	R					O **G^88^**	U	4		
**No. species per reef or quadrat**		**132**	**131**	**112**	**95**	**80**	**54**	**167**	**209**	**T=269**	**13 (7)**	**127 (93)**

**Notes relating to taxon names**: * Asterisk after species name indicates specimen and/or tissue specimen was cataloged in UMSNDH collection. **p** after species name indicates existence of published records of this species on the study reefs, all other species are new records. **1.** Chávez recorded *B.soporator*, from which *B.antillensis* has recently been split; *B.antillensis* is the insular form (L Tornabene personal communication to DRR 2019), hence we record only that species. **Habitat**: R = demersal on reef, SB = demersal on soft bottom, P = pelagic, BP = benthopelagic, C = cryptic, SC = semicryptic (more visible than cryptic species). **Ranked abundance on reefs** (see methods for details): A = abundant; C = common, widespread; LC = locally common; O = occasional; U = uncommon; R = rare; P = present but no data on abundance; blank = not recorded on a particular reef or quadrat. **No. reefs** = no. reefs on which a species was recorded; **2018 superscript**: species recorded at Cayo Arcas by us in 2018 but not 2016. **No. species per reef or quadrat**: T = total no. species recorded on reefs by us, Garduño (1988) or Chávez (1966); a (b) = total no. species in quadrat (no. species in quadrat that were not recorded by us on any reef). **C^66^**: species collected by Chávez (1966) at Cayo Arenas and Triangulo Oeste, but not recorded by us. **G^88^**: species observed by Garduño (1988) at Cayo Arenas.

### Species Notes

These notes refer to information relating to species of particular interest, e.g., those possibly misidentified or which can be confused with other similar species, invasive species, and species that are thought to be reliant on mangroves or seagrass beds for nursery habitats.

***Halichoeresburekae*** Weaver & Rocha, 2007 and ***H.pictus*** (Poey, 1860). *Halichoeresburekae*, a western Gulf of Mexico endemic, is perhaps the most abundant labrid on all eight reefs. This species is listed as Endangered (i.e., at a high risk of extinction) by the IUCN Red List (Rocha et al. 2015) due to the small size of its geographic range and the paucity of reef habitat within that area. The abundance of this species on the west Campeche reefs has substantial conservation significance, as it indicates that the set of small offshore reefs scattered along the western side of Campeche Bank may be essential for its continued existence. None of those reefs are as yet designated as Marine Protected Areas. There is a museum record of *H.pictus* in the Triángulo Quadrat, although we did not observe this species on any of our study reefs. This species is conspicuous and found on shallow fore-reefs, feeding in midwater in small aggregations in the same habitat as *H.burekae*, which also forms aggregations. Older records of this species on inshore reefs of Veracruz state, and (perhaps) Alacranes reef, may also relate to *Halichoeresburekae*, a recently described (2007) species endemic to that region, as the latter (but not *H.pictus*) is included in checklists from those reefs published after that date (e.g., González-Gándara 2014; González-Gándara et al. 2012, 2013, Aguilar-Perera and Tuz-Sulub 2009). *H.burekae* is abundant on the west Campeche reefs, and also common at Alacranes reef and the inshore reefs of Veracruz state.

***Halichoerespoeyi*** (Steindachner, 1867).This species typically is found in seagrass beds around the fringes of Caribbean Reefs. A single specimen of *H.poeyi* was collected at Cayo Arcas in 2018. However, it was not observed by DRR during either the 2016 or 2018 expeditions to that reef or any of the other seven reefs considered here. Hence it must be rare on those reefs. It is present on Alacranes reef (González-Gándara and Arias-González 2001), which has seagrass beds, and on the coastal reefs of Veracruz (del Morales-Flores et al. 2013) that also have adjacent seagrass beds.

***Xyrichtyssplendens*** (Castelnau, 1855). This species typically is found in seagrass beds adjacent to Caribbean reefs. On the west Campeche reefs we repeatedly observed small groups of adults associated with concentrations of macroalgae attached to small rocks in and around sandy areas on the leeward sides of reefs.

***Stegastesdiencaeus*** (Jordan & Rutter, 1897) and ***S.adustus*** (Troschel in Muller, 1865). *Stegastesdiencaeus* was not observed by DRR at Cayo Arcas in either 2016 or 2018, although we collected one individual there in 2018. This species was present, but uncommon in coralline-rock habitats at ~ 10–15m depth at both Cayo Arenas and Triángulo Oeste. As noted by Robertson et al. (2016b), records by Garduño (1988) of “*Eupomacentrusmellis*”, which is the juvenile of *S.diencaeus* (see Robertson and Allen 1981) on Cayo Arcas quite possibly relate to the similarly colored juveniles of *S.leucostictus* and *S.xanthurus*, which are easily confused with each other. Adults of *S.adustus*, which are similar in form and color to those of *S.diencaeus* and are easily confused with it (see Robertson et al. 2016b), were abundant on reef habitats with well-developed structure between 0.5–20 m on the five emergent reefs that had such habitat (i.e., all except the three Bancos).

***Neopomacentruscyanomos*** (Bleeker, 1856). This Indo-Pacific damselfish was locally common to abundant on all reefs we visited, and was observed in aggregations of up to ~ 100 individuals (Robertson et al. 2016a).

***Pteroisvolitans*** (Linnaeus, 1758). This species was uncommon in the shallow depths at which we dived at all the reefs visited between 2016 and 2018. At Cayo Arcas in 2016 the group of eight divers recorded <12 individuals (Robertson et al. 2016b). A similar number was seen by the group of ten divers who worked at 21 different sites there in 2018. During both the 2016 and 2018 visits a single adult was seen at ~ 30m depth on the oil loading platform 1.5 km from the Arcas reef. At Cayo Arenas the group of seven divers recorded a total less than ten individuals at 38 dive sites visited during May 2017. During the Triángulos trip in 2018 23 divers visited 59 dive sites and noted < 25 individuals in total. At Alacranes Reef on the center of Campeche Bank lionfish is uncommon in shallow water (DRR pers. obs. 2016), and is more abundant at depths below 30m (Aguilar-Perera et al. 2016).

***Sphyraenabarracuda*** (Edwards in Catesby, 1771). Adults of all sizes and medium to large juveniles were seen on all eight reefs, with large numbers of subadults (an aggregation of ~ 100 fish, each ~ 70 cm TL on one dive) in the lagoon of the lighthouse island of Cayo Arenas. A few small (3–5 cm) juveniles were observed associated with dislodged clumps of macroalgae on the sand bottom of the lagoon on the leeward side of the lighthouse cay at Cayo Arenas.

***Ocyuruschrysurus*** (Bloch, 1791). This was a common species on all reefs, and present in scores around the Cayo Arcas oil loading platform (see Robertson et al. 2016b). Adults and larger juveniles were both common, and small, recently recruited juveniles ~ 5 cm TL were seen in two situations: (i) a few single individuals were noted with dislodged macroalgae on the 3 m deep floor of the lagoon on the leeward side of the lighthouse island at Cayo Arenas; and (ii) dozens of individuals associated with each of several small (1–2 m diameter) patches of coral debris on sand at ~ 30 m depth on the seaward side of that same reef.

***Lutjanusapodus*** (Walbaum, 1792). Although this species was not observed at Cayo Arcas in 2016, several adults were noted in 2018. At Cayo Arenas in 2018 up to 30 adults of various sizes per dive were recorded on several dives. No small juveniles were seen at either site.

***Coryphopterushyalinus*** Böhlke & Robins, 1962 and ***C.personatus*** (Jordan & Thompson, 1905). These two small, hovering gobies are very similar in appearance, and are sufficiently similar that usually they are combined in faunal surveys. They were abundant in aggregations of up to hundreds of fish hovering within a meter of the bottom in sheltered habitats on the four emergent reefs abundant and present on the Bancos. Both species were collected at Cayo Arcas in 2018, and subsequently identified in the laboratory, and we assume both were present on the other reefs as well.

***Scarusguacamaia*** Cuvier, 1829. This species was seen on Cayo Arcas reef, in both 2016 and 2018, with ~ 20 small to large adults seen at various different sites on both occasions. However, because the surveys of each reef during the Triángulos expedition were relatively brief, and not specifically aimed at assessing this species, we are uncertain whether this species was present on any of the reefs examined during that expedition. No individuals of this species were seen at Cayo Arenas.

***Scaruscoeruleus*** (Bloch, 1786). This species was invariably observed over low-energy sandy bottoms, notably in the semi-enclosed lagoons on the western sides of reefs. It was common at Cayo Arcas, but none of the other reefs.

## Discussion

### Comprehensiveness of the faunal lists

Small cryptic fishes commonly represent ~ 40% of the reef-associated shorefish faunas in the neotropics and elsewhere (Smith-Vaniz et al. 2006, Robertson and Smith-Vaniz 2008, Brandl et al. 2018). The most effective way to comprehensively enumerate the cryptic component of the fish fauna of a reef is with the use of small amounts of anesthetics such as clove oil or quinaldine (Robertson and Smith-Vaniz 2010), and small ichthyocide stations (Smith-Vaniz et al. 2006, Robertson and Smith-Vaniz 2008). The latter generally are much more effective than anesthetics at dislodging fishes living deep within the matrices of coralline habitats and allow sampling of larger areas of reef in single stations: ~ 5–10 m diameter vs < 1 m diameter for anesthetics (Robertson and Smith-Vaniz 2008, 2010, and see Goatley et al. 2016). However, as in the present case, use of rotenone often is forbidden by permitting authorities and factors such as cost and local availability determine which anesthetic (clove oil, quinaldine, quinaldine sulphate, MS222) is used in any particular study. Use of clove oil produced specimens of 62 cryptic, reef-associated fish species from the eight reefs we sampled, 26% of the 237 demersal (non-pelagic) fishes we recorded. Cryptic species also represented 35 (74 %) of the 47 species added to the fauna of Cayo Arcas in 2018, and brought the representation of such species up to 24% (47) of the demersal fish fauna (199 species) on that reef. We note however, that the anesthetic stations employed were very small, each using ~ 100 ml of 10% clove oil solution delivered by squirt-bottle that affected much less than 0.25 m^2^ of the surface substratum and superficial crevices. This sampling was aimed primarily at obtaining specimens for a study of connectivity among reef fish populations of the southwest Gulf of Mexico and Caribbean Mexico. Due to time limitations the full variety of types of hard-reef microhabitats and soft-sediment microhabitats within and bordering reef areas were not comprehensively sampled. Hence full documentation of the cryptic reef-fish fauna of these reefs has yet to be done. For example, we collected no ophichthid or congrid eels, no antennariids, gobiesocids, or callyonymids, only a single syngnathid, and no cuskeels. Thus 24% cryptic species likely is an underestimate of that component of the Cayo Arcas demersal fish fauna. For Cayo Arenas, a reef complex of similar size and form to that at Cayo Arcas, 167 species also seems low, especially given that only 14% (24) of the species currently recorded from there are cryptic. For Triángulo Oeste, the reef most heavily sampled during the 2018 cruise of the *Isla Mujeres*, the cryptic representation was 31% (41) of the 132 species, although the total also seems low, even for reef much smaller than the reefs at either Cayo Arcas or Cayo Arenas. Clearly there is more collecting to be done before the reef-fish fauna of this area can be classed as comprehensively sampled.

### Common species on the study reefs: 1980s vs. 2010s

Table 2 includes information on the relative commonness of species at Cayo Arcas and Cayo Arenas during either or both of two periods separated by 30+ years. This list includes species defined as numerically dominant (ie common) at one or both reefs in the 1980s by Garduño and Chávez (2000) and others that were common to abundant during our surveys in 2016–18 but not present in the 1980s list. Among the 42 common 1980s species only seven were relatively uncommon in the 2010s: *Lutjanusapodus*, *Haemulonplumieri* (Lacepède, 1801) *H.sciurus* (Shaw, 1803) and *Holacanthustricolor* (Bloch, 1795) at both reefs, and *Anisotremusvirginicus* (Linnaeus, 1758), *Brachygenyschrysargeum* (Günther, 1859), and *Haemuloncarbonarium* Poey, 1860 at one reef. In contrast 17 species that were common in the 2010s were not listed among the 1980s group. All species on both lists, except *Halichoeresburekae*, are common and widely distributed inhabitants of Greater Caribbean reefs. Some, but not all, of the differences between the two lists can be attributed to the use of different methodologies: small transects used in the 1980s (see Garduño and Chávez 2000) are less likely to detect locally abundant and patchily distributed species than are the wide-ranging “seascape” visual surveys by us in the 2010s that sampled much larger areas. Exceptions include all seven species common in the 1980s but not the 2010s. Fishing is an obvious factor to consider with most of those seven, particularly species like *L.apodus*. While we often saw small fishing boats around the reefs during our expeditions they fished during the day several kilometers or more away from the reefs and only came in to shelter at the leeward edges of reefs at night. We saw no fish traps during any of our diving surveys, and the fishing boats were relying on hook-and-line fishing. In addition, the Cayo Arcas reef-system derives a measure of protection from fishing by being in a security exclusion zone, due to its close proximity to major offshore oil installations. However, *L.apodus* is a nocturnally active species that uses shallow reef habitats as resting sites during the day and migrates distances of up to at least several km away from those reefs to feed at night (Nagelkerken 2009, Hitt et al. 2011, Friedlander et al. 2013). Such diurnal migrations could make it susceptible to off-reef fishing, which could affect populations sheltering on small reefs (cf. Halpern 2004). Differences in methodology could account for some of the species that were common in the 2010s not being so in the 1980s. However, there are some obvious exceptions to that possibility: *Stegastesadustus*, *H.burekae*, *Scarusiseri* (Bloch, 1789) and *Sparisomaaurofrenatum* (Valenciennes in Cuvier and Valenciennes, 1840) all of which are common throughout a wide range of habitats today and are susceptible to being counted in transects. *Halichoeresburekae* was exceptionally abundant and widely distributed across a range of habitats in the 2010s. Although this species was not described and named until 2007 there are no references made to any species similar to it (e.g., *Halichoerespictus*) in the 1980s. There is no obvious reason why there was no mention of these four species in the 1980s.

**Table 2.** Relative abundance of fishes classified as dominant species on Cayo Arcas and Cayo Arenas during 1984–86 by Garduño (1988) as reported in Chávez and Beaver (2007) and our own observations on the same reefs in 2016–18.

Species	Arenas 2017	Arenas 1980s	Arcas 2016–18	Arcas 1980s
**HOLOCENTRIDAE**				
*Holocentrusrufus* *	C	YES	O	YES
**SERRANIDAE**				
*Cephalopholiscruentata*	C		C	
*Cephalopholisfurcifer*	LC		C	
*Epinephelusadscensionis*	O		C	
*Epinephelusguttatus* *	O	YES	C	YES
*Serranustigrinus* *	C	YES	C	YES
**GRAMMATIDAE**				
*Grammaloreto* *	LC	YES	C	YES
**CARANGIDAE**				
*Caranxruber*	LA	YES	LC	YES
**LUTJANIDAE**				
*Lutjanusapodus*	O	YES	U	YES
*Lutjanusgriseus* *	C		C	
*Lutjanusmahogoni*	C		O	
*Ocyuruschrysurus* *	A	YES	A	YES
**HAEMULIDAE**				
*Anisotremusvirginicus*	–	YES	LC	YES
*Brachygenyschrysargyreum* *	–	YES	LC	YES
*Haemulonaurolineatum* *	LC	YES	C	YES
*Haemuloncarbonarium*	R	YES	U	YES
*Haemulonflavolineatum* *	C	YES	C	YES
*Haemulonplumierii* *	-	YES	U	YES
*Haemulonsciurus*	R	YES	U	YES
*Haemulonvittatum* *	C	YES	LC	YES
**MULLIDAE**				
*Mulloidichthysmartinicus* *	C		C	YES
**PEMPHERIDAE**				
*Pempherisschomburgkii* *	LC	YES	LC	
**KYPHOSIDAE**				
*Kyphosussectatrix*	C		C	
*Kyphosusvaigiensis*	C		C	
**CHAETODONTIDAE**				
*Chaetodonocellatus*	C		C	
*Chaetodonsedentarius*	C		C	
**POMACANTHIDAE**				
*Holacanthustricolor* *	R	YES	R	YES
*Pomacanthusparu* *	O	YES	LC	YES
**POMACENTRIDAE**				
*Abudefdufsaxatilis* *	A	YES	A	YES
*Chromiscyanea* *	LC	YES	LC	YES
*Chromismultilineata* *	A	YES	A	YES
*Chromisscotti*	C		C	
*Microspathodonchrysurus* *	C	YES	C	YES
*Stegastesadustus*	A		A	
*Stegastesleucostictus* *	LC		LC	
*Stegastespartitus* *	A	YES	A	YES
*Stegastesplanifrons* *	O	YES	A	YES
*Stegastesxanthurus* *	C	YES	A	
**LABRIDAE**				
*Clepticusparrae* *	A	YES	LC	YES
*Halichoeresbivittatus*	A		A	
*Halichoeresburekae*	A		A	
*Halichoeresgarnoti* *	C	YES	C	YES
*Halichoeresmaculipinna* *	A	YES	C	YES
*Halichoeresradiatus* *	–		C	
*Thalassomabifasciatum* *	A	YES	A	YES
**SCARIDAE**				
*Scaruscoeruleus* *	U	YES	C	YES
*Scarusiseri*	C		C	
*Scarustaeniopterus* *	C	YES	C	YES
*Scarusvetula* *	A	YES	C	YES
*Sparisomaaurofrenatum* *	LC		C	
*Sparisomachrysopterum*	A	YES	O	YES
*Sparisomarubripinne*	LC		LC	YES
*Sparisomaviride* *	C	YES	C	YES
**ACANTHURIDAE**				
*Acanthuruschirurgus*	C	YES	C	YES
*Acanthuruscoeruleus* *	A	YES	C	YES
*Acanthurustractus* *	A	YES	C	YES
**SPHYRAENIDAE**				
*Sphyraenabarracuda* *	A		O	
**BALISTIDAE**				
*Melichthysniger*	LC	YES	LC	
**TETRAODONTIDAE**				
*Canthigasterrostrata* *	C	YES	C	YES

Notes: * Asterisk after species name indicates specimen and/or tissue specimen was cataloged in UMSNDH collection. **Habitat**: R = demersal on reef, SB = demersal on soft bottom, P = pelagic, BP = benthopelagic, C = cryptic, SC = semicryptic (more visible than cryptic species). **Ranked abundance on reefs (see methods for details)**: A = abundant; C = common, widespread; LC = locally common; O = occasional; U = uncommon; R = rare; P = present but no data on abundance; blank = not recorded on a particular reef or quadrat. **No. reefs** = no. reefs on which a species was recorded by us; **2018 superscript**: species recorded by us in 2018 but not 2016. **No. species per quadrat**: a (b) = total no. species in quadrat (no. species in quadrat that were not recorded by us on any reef).

### “Mangrove/seagrass nursery species” of reef fishes at isolated Greater Caribbean reefs that lack adjacent nursery habitat(s)

A series of studies have developed around the hypothesis that certain common species of reef fishes in the Greater Caribbean use mangroves and seagrass beds as nurseries (hereafter nursery-species) and are sufficiently reliant on one or both of those as nursery habitats that their local abundances reflect the local availability of those nursery habitats adjacent to reefs (e.g., Nagelkerken et al. 2000, 2017, Serafy et al. 2003, Dorenbosch et al. 2004, 2006, 2007, Halpern 2004, Mumby et al. 2004, Scharer et al. 2007, Scharer 2009, Jones et al. 2010, Claydon et al. 2015). Those studies have relied primarily on examination ofreef areas that contain such habitats, in either variable amounts or at varying distances from local reefs within large reef complexes. However, a few studies have examined nursery-species at locations that lack mangroves but have seagrass beds (Scharer 2009; Aguilar-Perera and Hernández-Landa 2017).

Below we summarize information on the occurrence and, in some cases, abundance of 16 species of reef-fishes commonly regarded as nursery-species in the faunas of various isolated Caribbean reefs that lack mangroves, and, in some instances, seagrass beds. Campeche Bank offshore reefs are among them.

**Campeche outer bank reefs.** Table 3 contains information on the abundances of nursery-species on West Campeche reefs (no mangroves or seagrasses) and Alacranes reef (seagrasses only). Of those, all are common on Alacranes except *Lutjanusapodus* and *Scarustaeniopterus* (Lesson in Bory de Saint-Vincent, 1829). Eight species are common on West Campeche reefs, including *Scarustaeniopterus*, one (*Haemulonparra* (Desmarest, 1823)) is absent on West Campeche reefs and the remaining six are uncommon. The vast area (~ 100,000 km^2^) of Campeche bank is relatively shallow, with depths of 30–50 m in most parts. Inter-reef areas comprise a mixture of soft bottoms and small patches of coral and sponges (Hedgpeth 1954, Logan et al. 1969). Bycatch from shrimp trawlers working on soft bottoms on the part of Campeche bank south of the study reefs that were recorded by Hildebrand et al. (1964) included three of the 16 nursery species: *Lutjanusgriseus* (Linnaeus, 1758), *Ocyuruschrysurus*, and *Haemulonplumieri*.

**Table 3.** Sixteen species of common Greater Caribbean reef-fishes thought to be reliant on mangroves and seagrass beds as near-reef nursery habitats, and their general abundance on the west Campeche study reefs and Arrecife Alacranes.

Species	Mangroves	Seagrass	W Campeche	Alacranes
Mangrove & seagrass present?			Neither	Seagrass
*Lutjanusanalis*	++	++	Rare	Common
*Lutjanusapodus*	++	+	Occasional	Uncommon
*Lutjanusgriseus*	++	++	Common	Common
*Lutjanusmahogoni*	++	++	Locally Common	Common
*Ocyuruschrysurus*	++	++	Abundant	Abundant
*Haemulonflavolineatum*	++	++	Common	Common
*Haemulonparra*	++	+	Absent	Common
*Haemulonplumieri*	++	++	Occasional	Common
*Haemulonsciurus*	++	++	Uncommon	Common
*Chaetodoncapistratus*	++	+	Occasional	Common
*Scaruscoeruleus*	+	+	Locally common	Common
*Scarusguacamaia*	++		Uncommon	Common
*Scarusiseri*	+	++	Common	Abundant
*Scarustaeniopterus*	++	+	Common	Uncommon
*Acanthuruschirurgus*	+	+	Common	Common
*Sphyraenabarracuda*	++	+	Common	Common

**Notes**: ++ indicates strong dependency, + weaker dependency. Sources: Usage of seagrass and mangroves as reef fish nurseries: Nagelkerken et al. 2000a, b, 2001, 2017; Cocheret de la Morinière et al. 2002; Nagelkerken and van der Velde 2003; Halpern 2004; Mumby et al. 2004; Dorenbosch et al. 2004, 2006, 2007; Verweij et al. 2008; Nagelkerken 2009; Scharer 2009, Machemer et al. 2012, Harborne et al. 2015, Serafy et al. 2015. Claydon et al. 2015. W Campeche: a summary of results presented here. Fishes of Alacranes reef: González-Gándara and Arias-González 2001, abundance based on observations by DRR during dives at 23 different sites at Alacranes reef during May 2016.

**Veracruz (Mexico) coastal reefs.** Published checklists are available for seven coastal reefs in the northern part of Veracruz state. Of those reefs six are emergent and one submerged, none have mangroves and only two of the emergent reefs have seagrass beds (Table 4). Those reefs vary in their degree of isolation from the mainland coast and from each other. Mexican government chart SM 030 indicates those reefs are all on the continental shelf in water less than ~ 50 m deep, 5–20 km from the coast. The nature of the inter-reef bottoms in that area is unclear. González-Gándara (2014) used an extensive set of surveys to define the fish fauna of Blake Reef, a small (2.5 km long) submerged (minimum depth 9 m) reef that is isolated from both the shore and emergent reefs (20 km from the shore, 36 km from the nearest emergent reef). That reef lacks both seagrasses and mangroves, and the top surface is a plain covered with boulders, corals and sponges (C González-Gándara pers. comm. to DRR 2018). Of the 16 nursery species, only four are not listed at Blake Reef (Table 4). On the six emergent reefs (González-Gándara et al. 2012, 2013) the only nursery species that were absent on all but one reef were *Haemulonparra* and *H.sciurus*.

**Table 4.** Sixteen mangrove/seagrass nursery-fishes present at reefs lacking mangroves, and, in some cases, seagrasses, on the continental shelf near Tuxpan, Veracruz, Mexico.

Species	Lobos	Medio	Blanquilla	Blake	Tanhuijo	Enmedio	Tuxpan
Submerged/Emergent	Emergent	Emergent	Emergent	Submerged	Emergent	Emergent	Emergent
Mangrove/Seagrass	No/Yes	No/No	No/No	No/No	No/No	No/No	No/Yes
Distance from mainland (km)	11.5	7.5	5	20	10	10	13
Fish Species							
*Lutjanusanalis*					Yes	Yes	Yes
*Lutjanusapodus*	Yes	Yes	Yes	Yes		Yes	Yes
*Lutjanusgriseus*	Yes	Yes	Yes	Yes	Yes	Yes	Yes
*Lutjanusmahogoni*	Yes		Yes	Yes	Yes	Yes	Yes
*Ocyuruschrysurus*	Yes	Yes	Yes	Yes	Yes	Yes	Yes
*Haemulonflavolineatum*	Yes	Yes	Yes	Yes	Yes	Yes	Yes
*Haemulonparra*			Yes				
*Haemulonplumieri*	Yes	Yes	Yes	Yes	Yes	Yes	Yes
*Haemulonsciurus*					Yes		
*Chaetodoncapistratus*	Yes	Yes		Yes	Yes	Yes	Yes
*Scaruscoeruleus*	Yes	Yes			Yes		
*Scarusguacamaia*	Yes	Yes		Yes		Yes	Yes
*Scarusiseri*	Yes	Yes	Yes	Yes	Yes	Yes	Yes
*Scarustaeniopterus*	Yes	Yes	Yes	Yes	Yes	Yes	Yes
*Acanthuruschirurgus*	Yes	Yes	Yes	Yes	Yes	Yes	Yes
*Sphyraenabarracuda*	Yes		Yes	Yes	Yes	Yes	Yes

**Sources**: González-Gándara et al. 2013, González-Gándara 2014

**Flower Garden Banks.** These banks are two submerged patches of coral reef located 180 km offshore from the coast of Texas, on the continental shelf. Minimum depth is 17 m, the banks are surrounded by water >50 m deep, and there are no seagrasses or mangroves. Muñoz et al. (2017) found seven nursery species present during quantitative surveys, four of them moderately common (Table 5). For most of the nursery species not recorded by those authors or by the Flower Garden Bank MPA website, these banks were either out of or at the latitudinal limit of their geographic range, and hence the species would not likely be common enough to be registered by Muñoz et al. (2017)

**Table 5.** Occurrence of 16 mangrove/seagrass nursery-fishes at isolated, emergent, and submerged reefs in the northern Gulf of Mexico and the Caribbean that lack mangroves, and, in some cases, seagrass nursery habitats.

Species	Mona Island	Swan Island	Flower Garden Banks	Saba Bank	Navassa Island
On continental shelf?	No	No	Yes	No	No
Isolation distance (Km)	60	170	180	30*	35
Submerged/Emergent	Emergent	Emergent	Submerged	Submerged	Emergent
Mangrove/Seagrass	No/Yes	No/No	No/No	No/No	No/No
Fish Species					
*Lutjanusanalis*	Uncommon		(Out of range)		
*Lutjanusapodus*	Common	Present	Present (Limit of range)	Present	Common
*Lutjanusgriseus*	Uncommon		Common		
*Lutjanusmahogoni*	Common	present	Uncommon (Limit of range)	Common	
*Ocyuruschrysurus*	Uncommon	present	Uncommon	Present	Uncommon
*Haemulonflavolineatum*	Common	Present	(Out of range)	common	Uncommon
*Haemulonparra*	Common		(Limit of range)		
*Haemulonplumieri*	Uncommon	present	Uncommon	Common	
*Haemulonsciurus*	Uncommon	Present	(Out of range)		Uncommon
*Chaetodoncapistratus*	Common	Present	(Limit of range)	Present	Uncommon
*Scaruscoeruleus*			(Out of range)		Uncommon
*Scarusguacamaia*	Present	Present	(Out of range)	Present	
*Scarusiseri*	Common	present	Common (Limit of range)	Common	Uncommon
*Scarustaeniopterus*	Common	present	Common (Limit of range)	Common	Uncommon
*Acanthuruschirurgus*	Uncommon	present	Common	Common	Uncommon
*Sphyraenabarracuda*	Uncommon	present	Present	Common	Common

**Sources**: Mona – Scharer (2009) and see species account; Swan – Aggra (2013); Flower Garden Banks – Muñoz et al. (2017) and https://flowergarden.noaa.gov/about/fishlist.html; Navassa – McClellan & Miller (2002), Collette et al. 2003; Saba – Toller et al. 2010 (abundance data), Williams et al. 2010 (presence/absence)(* Saba Island, 6 km from, and separated by several kilometers of very deep water from Saba Bank, lacks mangroves and *Thalassia*, and the nearest location with *Thalassia* is St. Eustatius island, 30 km from that bank). Out of range: site is outside the geographic range of the species. Limit of range: site is at or near latitudinal limit of the geographic range of the species.

**Navassa Island**. This 3.5 km-long island has a narrow fringing reef and rises abruptly out of deep water between Haiti and Jamaica. It is 57 km from land and separated from the shelf around Hispaniola by 35 km of deep (>1000 m) water. Almost the entire reef is 25 m or deeper. Navassa has no mangroves or seagrass beds, but does have substantial stands of macroalgae. The island’s limited reef area likely is overfished by subsistence fishers from Haiti (Miller 2002, Sandin 2002). Ten nursery species are present at the island, most of them common (Table 5). Although there are few haemulids and lutjanids at this island, two nursery species dominated the biomass of carnivores at the beginning of the 2000s: *Lutjanusapodus* and *Sphyraenabarracuda*.

**Mona Island.** Mona Island is a 10 km long island that arises precipitously out of deep water, has a narrow shallow fringing reef, no mangroves in the sea and only ~ 1 km^2^ of seagrass, in beds or mixed with rubble, corals, bedrock and sand patches (Scharer 2009). Located in the channel between Hispanola and Puerto Rico, this island is separated by 60–70 km from those two large mangrove bearing islands. Scharer (2009) examined habitat usage and various aspects of the ecology of the reef-fish fauna, focusing in particular on ontogenetic changes in habitat usage by nursery species. Of the 16 nursery species, seven were common, seven were uncommon, and two were absent (Table 5). Abundances of three nursery species are of particular interest: *Lutjanusapodus* and *Lutjanusmahogoni* (Cuvier in Cuvier and Valenciennes 1828) were common, and *Haemulonparra* was moderately common (Scharer 2006). Scharer (2009) and Scharer et al. (2007) found that nursery habitat usage by eight common species for which sufficient data were available for analysis had the following characteristics: nursery habitats typically were shallow; the smallest juveniles were concentrated in seagrass habitats, although most also used hard bottoms; juveniles expanded the range of nursery habitats they used as they grew.

**Saba Bank.** This large (2,200 km^2^) submerged coralliferous bank that lacks both seagrass and mangroves is separated by a narrow (several km) stretch of deep water from Saba Island, 6 km away. Minimum depth of the bank is ~ 11 m. There are no mangroves on Saba Island and the only seagrasses there are small patches of *Syringodium*. Toller et al. (2010) and Williams et al. (2010) documented the reef-fish fauna of Saba bank, where 11 nursery-species are present, six of them common (Table 5).

**Swan Islands.** This doublet of islands is situated in deep water 170–180 km offshore from Honduras and the nearest emergent reefs. The area of shallow reef is ~ 8X3 km, and neither island has mangroves. Whether or not there are seagrasses is unclear. The only known survey of the reef-fishes of that island is by AGRRA (http://www.agrra.org/), which uses counts of fishes on small transects, which are likely to miss large, mobile, wide ranging species that avoid divers. That survey, which was made in 2013, and is far from complete, listed 64 species, including 12 of the 16 nursery species (Table 5).

### Individual nursery-species accounts (composites from island-fauna accounts)

**Lutjanidae.***Lutjanusanalis* (Cuvier in Cuvier and Valenciennes, 1828) uses a variety of habitats as nursery habitat (Lindeman et al. 2016a), in addition to mangrove and seagrasses. It varies from being absent to common on reefs lacking nearby mangroves (Tables 3–5), and generally is rare to absent on reefs without both mangroves and seagrass beds, indicating it may well be dependent on such habitats as nurseries. Juveniles of *L.apodus*, another “mangrove-dependent” nursery species, also use rocky habitat as nursery (Lindeman et al. 2016b). It is sometimes common at sites without mangrove that have seagrasses, which also is used as nursery habitat (Hildebrand et al. 1964), and can be present in significant numbers at sites without either habitat (Tables 3–5). Halpern (2004) suggested that the population at Navassa Island was maintained by immigration from Haiti. However, this is extremely unlikely given the large distances involved; the fact that *Lutjanusapodus* is a demersal species not known to extend below 156 m, and that most of the distance between Hispaniola and Navassa is very deep water. *Lutjanusgriseus* is another nursery species, the juveniles of which also use estuaries (Lindeman et al. 2016c). It is common at isolated reefs on the continental shelf that lack mangroves, and in some cases, seagrasses, but is absent on most isolated oceanic reefs lacking such habitat (Table 3–5). Which habitats are crucial for producing this distribution pattern is far from clear. *Lutjanusmahogoni* is thought to be weakly dependent on nursery habitats, and can be common at sites without either nursery habitat. *Ocyuruschrysurus* uses a variety of microhabitats as nurseries, including hard bottom, in addition to mangroves and seagrass beds (Lindeman et al. 2016d, Hildebrand et al. 1964). Large areas of inter-reef substrata scattered over the 100,000+ km^2^ of Campeche Bank that have small patches of rubble microhabitat we saw used by this species as nursery could sustain large populations of this species on the small shallow and emergent reef areas lacking mangroves or seagrasses along the western edge of Campeche Bank. Lutjanids are also known to migrate appreciable distances over shallow shelf habitats, as much as 65 km in the case *L.griseus* (see Nagelkerken 2009). Such relocation across shallow shelf areas, particularly those with stepping-stone patches of submerged reefal habitat, such as sponges (Hedgepeth 1954, Hildebrand et al. 1964), could account for populations of this species on on-shelf reefs far from nursery habitat, at least 180 km in the case of the Flower Garden Banks. Known diel movements of *L.apodus* and *L.analis* to off-reef habitats from daytime resting areas on emergent reefs are much shorter, on the order of < 10 km (Hitt et al. 2011, Friedlander et al. 2013), but a capacity for such activity could be sufficient to provide connectivity across shallow shelves that have scattered patches of submerged reefal habitats (e.g., sponge beds), or increase the susceptibility of fish observed on reefs during the day to fishing some distance away from those reefs.

**Haemulidae.***Haemulonflavolineatum* (Desmarest, 1823) is common on all reefs within its geographic range, regardless of the mangrove/seagrass status of those reefs (Tables 3–5). *Haemulonparra*, which is classed as seagrass-dependent for nursery habitat (and see Hildebrand 1964), appears to be one of the few species that typically is absent on reefs lacking such habitat (Tables 3–5). *Haemulonplumieri* has been classed as mainly mangrove dependent (Table 3), but commonly uses shallow hard bottoms and seagrass beds as nurseries (Lindeman et al. 2016e). It is found, often commonly, on reefs without either mangroves or seagrass beds. *Haemulonsciurus* has been classed as mainly mangrove-dependent, but also using seagrass and hard-bottoms as nursery habitat (Table 3). It is present on reefs lacking mangroves, but uncommon to absent on those without seagrass beds (Tables 3–5). Information available on the mobility of grunts such as *H.flavolineatum*, *H.plumieri*, and *H.sciurus*, indicates that they range over relatively short distances, < 5 km (Friedlander et al. 2013). Whether or not this capacity for mobility is sufficient to allow grunts to move across large distances of shelf between the shore and isolated on shelf reefs is unclear. Perhaps it does for *H.plumieri*, which is trawled in inter-reef areas on Campeche Bank where unnamed, submerged coral and sponge patches are common.

**Chaetodontidae.***Chaetodoncapistratus* Linnaeus, 1758 has been classed as mangrove-dependent (Table 3), but is present, sometimes common, on reefs without mangroves, and, in some cases, without seagrasses. This species evidently is capable of maintaining significant local populations using other nursery habitats.

**Scaridae.***Scaruscoeruleus* has sometimes been classed as mangrove/seagrass nursery dependent (Table 3). Its adults typically feed on low-energy sandy bottoms (Rocha et al. 2012, DRR pers. obs.). It does occur, sometimes commonly, on isolated reefs lacking one or both nursery habitats, and its occurrence may also be influenced by the availability of suitable sandy habitat for adults.

The Rainbow parrotfish, *Scarusguacamaia*, which reaches 120 cm TL, is the largest parrotfish in the Greater Caribbean. It is typically observed in small groups or schools that roam over large areas (Mumby et al. 2004). It is typically seen feeding in shallow reef-edge habitats, including intertidal areas, in water < 5 m deep (Claydon et al. 2015, Hernández and Aguilar-Perera 2018, DRR pers. obs.), although its depth range extends down to at least 55 m (MT Scharer pers. comm. December 2018). This iconic reef-fish is thought to be strongly reliant on mangroves as nursery habitat (Mumby et al. 2004, Claydon et al. 2015, and see studies cited in Table 2), to the extent that removal of mangroves can result in local extinction (Mumby et al. 2004).

Various studies of the habitat distributions of different size classes of *S.guacamaia* elsewhere have indicated that (i) juveniles observed in mangroves usually are ~ 10–20 cm (range 5–60 cm)TL (Dorenbosch et al. 2006; Nagelkerken and Van der Velde 2003, Nagelkerken 2009, Sefay et al. 2003, Jones et al. 2010, Claydon et al. 2015) and that the smaller numbers of juveniles seen in seagrasses near mangroves are somewhat larger than those in mangroves (Nagellkerken et al. 2002, 2009), indicating a shift from mangroves to seagrasses during development. None of those papers provided information on nursery habitats used by juveniles smaller than ~ 5 cm. More recently, however, Aguilar-Perera and Hernández-Landa (2017) observed juveniles 5–10 cm TL in shallow coral-rubble habitat on the reef top at Alacranes reef on Campeche bank, as well as adults and substantial schools of large juveniles on that reef. Those authors pointed out that juvenile *Scarusguacamaia* are not known from the nearest mangroves along the Gulf coast of the Yucatán peninsula, that the nearest coral reefs with adjacent mangroves are 300 km away. They proposed that the Alacranes population of this species is self-sustaining in the absence of mangrove habitat. Although, as noted above, there is one small patch of mangroves associated with a pond at the southeast corner of Isla Perez at Alacranes reef (Tunnell and Chapman 2001), due to its tiny size (~ 0.1 ha) and semi-isolated status that patch is unlikely to act as a significant piece of reef-fish nursery habitat. Long before Aguilar-Perera and Hernandez-Landa’s (2017) observations, Hildebrand et al. (1964) collected even smaller juveniles (3–4 cm) of this species in the extensive *Thalassia* beds at Alacranes reef using push nets, and considered those beds to be nursery habitat for this species. These two studies show that, on mangrove-free reefs, adequate nursery habitats for *S.guacamaia* can be present in the form of seagrass beds for the smallest juveniles after pelagic larvae recruit there, with shallow rocky habitats acting as secondary nursery habitat for somewhat larger juveniles that typically are seen in mangroves at other locations.

Claydon et al. (2015) examined the distribution of *S.guacamaia* on shoreline reefs at Bonaire, under the assumption that use of mangrove nurseries is obligatory for this species and the only nursery habitat of importance at that island. They found substantial densities of adults as much as 42 km from the nearest mangroves and assumed they had migrated such distances along the continuous shallow coastline reef. They also found very low densities of this species at a small, mangrove-free island (Klein Bonaire) separated from Bonaire by < 1km of water that has minimum depths of 80 m, and concluded that this was due to lack of immigration from Bonaire.

This species is now known to occur in appreciable numbers at a variety of isolated sites that lack mangroves and, in some cases seagrasses. This includes Cayo Arcas (150 km from the mainland shore), which, like other small, west Campeche emergent reefs, has many reef habitats found on Alacranes reef. If the population of *S.guacamaia* at Alacranes is self-recruiting then the assumption that that is the case with the Cayo Arcas population is reasonable. The small size of individual West Campeche reefs could make it difficult to sustain populations of large, low density species like *S.guacamaia*, and account for the apparent absence of this species on Cayo Arenas. The alternative to juvenile recruitment onto Cayo Arcas would be very long-distance migration, as the nearest reefs with adjacent mangroves are 350 km away in Veracruz state, while Alacranes reef is 330 km from Cayo Arcas, and 170 km from Cayo Arenas.

*Scarusguacamaia* also occurs on other emergent and submerged reefs lacking mangroves and, in some cases, seagrasses that are situated on the continental shelf but located away from the shoreline in Veracruz state, in the southwest Gulf of Mexico and in Venezuela. In Venezuela this species occurs at three archipelagos of small, rocky islands that lack structural coral reefs and mangroves, and in some cases seagrasses, that are found on the continental shelf off the coast of Venezuela. At each archipelago small groups (~ 6 fish) of adults were seen by DRR at multiple dive sites: Los Monjes at the mouth of the Gulf of Venezuela, 35–40 km from the shore of the Guajira Peninsula Colombia (DRR pers. obs. 2008), Los Frailes, 13 km from mangrove-bearing Isla Margarita (DRR pers. obs. 2005), and Los Testigos, ~ 70 km from both Los Frailes and the shore of the Paria Peninsula (DRR pers. obs. 2006). Depths of the shelf between those islands and the mainland are ~ 30–50 m. While at Isla Margarita in 2005 DRR saw a large adult (~ 1 m TL) of *S.guacamaia* that had been freshly caught be a shrimp trawler in shallow water ca. 1.5 km offshore from that island. This indicates that adults of this species do sometimes move through inter-reef areas of soft bottoms, although how far from reef habitat that individual was caught is not clear. The known depth range of *S.guacamaia* extends down to 55 m (MT Scharer, pers. comm. to DRR, December 2018). Hence, while it is feasible for a large species like *S.guacamaia* to have migrated to those isolated reefs across shallow shelf areas that seems unlikely: it would require that a species that prefers very shallow coral-reef habitat disperses tens of kilometers across unusable habitat and does so in large enough numbers for appreciable numbers of fish to find their way to tiny, isolated patches of non-coral habitat: the Los Monjes islands are all <1 km in diameter, the Frailes < 2 km, and the Los Testigos all < 5 km in maximum dimension. Larval recruitment to those islands, and to all other similarly isolated islands and reefs on the continental shelf that lack nearby mangroves seems much more likely.

Migration from sites that have nursery habitats to reefs isolated by deep water is even less likely than long-distance trans-shelf movements with larval recruitment the most likely source sustaining populations at such sites. *Scarusguacamaia* also is now known from sites scattered around the Caribbean that lack mangroves and, in some cases, seagrass beds and are isolated by deep water from the shelves of the nearest land that has such habitat: While there were no *Scarusguacamaia* in transect surveys made by Scharer et al. (2007) at Mona Island, fishers previously speared this species in shallow water there (M Scharer pers. comm. December 2018), and it has been observed at Monito Island, a 0.5 km diameter islet separated from Mona by 5 km of water ~ 50m deep. It also occurs at nearby Isla Desecho, which lacks both mangroves and seagrasses and is isolated by deep water, 40 km from Puerto Rico, and at Bajo Sico (neither nursery habitat), a submerged bank that rises to within 20m of the surface and is isolated by a 5 km stretch of 190 m-deep water from the shallows of the Puerto Rico shelf, and is 27 km from the nearest mangroves on that island (MT Scharer pers. comm. to DRR, December 2018). Other isolated sites in deep water that lack mangroves and seagrasses and at which this species is now know include Navassa Island and Saba Bank, as well as Swan Island, which lacks mangroves. *S.guacamaia* is listed as Near Threatened by the IUCN Red List (Choat et al. 2012), due in part to loss of mangrove habitat throughout its geographic range. However, while recruitment to non-mangrove habitats has been established, and is sufficient to maintain a substantial population on a large reef like Alacranes reef, the general significance of such an ability for maintenance of populations of this species are unknown. Large-bodied parrotfishes such as this occur at much lower population densities than small-bodied species and large areas of habitat for both juveniles and adults likely are necessary for maintaining isolated populations. Alacranes reef, which has a surface area of ~ 300 km^2^, provides such an area.

According to Mumby et al. (2015) the extinction of *S.guacamaia* on Glovers Reef (a 350 km^2^ atoll isolated by 20 km of deep water from the shelf edge Barrier Reef of Belize) in the 1970s was most probably due to the removal of mangroves there, although the species also was heavily fished during the mangrove-removal period. This atoll has huge areas of seagrass (>100 km^2^; Strindberg et al. 2016). However, the atoll has only five tiny (combined area <25 ha) sand cays, some of which once supported mangroves and the total area of mangroves prior to their removal to facilitate human habitation on several of those cays must have been tiny. Censuses in 2007 and 2017 indicate that this species has subsequently remained rare on that reef (A Tewfik, pers. comm. to DRR January 2019). Given what we now know about the ability of the rainbow parrotfish to maintain a population on mangrove-free Alacranes reef by using seagrass and rubble banks as nursery habitat, overfishing of a once self-recruiting population seems more likely than mangrove-loss to account for its demise and subsequent rarity.

To date studies of the relationship between *S.guacamaia* populations and abundance of nursery habitat have focused largely on mangroves as nurseries, and been based on observations alone (Mumby et al. 2004, Machemer et al. 2012, Claydon et al. 2015, and see other studies cited in Table 3). Future studies of nursery habitats of this species necessarily should involve examination and active collections aimed at small juveniles hidden in seagrass beds (cf. Hildebrand et al. 1964), and include observations and collections in other habitats, such as shallow rubble banks now known to be used by small juveniles. Comparison of densities of *S.guacamaia* in areas with and without seagrasses and mangroves are also needed, to assess the population impact of such nursery habitats, taking into account the likely effects of reef-size, and the extent of preferred, emergent habitat for adults on sizes of populations of a large, highly mobile, low density species. Correlational studies focused on the relationship between variation in abundances of mangroves and *S.guacamaia* at the regional level (e.g., Serafy et al. 2015) need to be revisited, incorporating variation in the abundance of both seagrasses and mangroves.

*Scarusiseri* has been classed as strongly dependent on seagrasses for nursery habitat (Table 3, and see Rocha et al. 2012, Hildebrand et al. 1964). It is ubiquitous, and typically common, on isolated reefs regardless of the presence or absence of both nursery habitats. *Scarustaeniopterus*, which is thought to be somewhat dependent on both mangroves and seagrass beds (Table 3), also is ubiquitous, and often common, on isolated reefs in the region that lack mangroves, and often seagrass beds. These two small parrotfishes evidently maintain significant local populations without mangroves or seagrasses. What habitats they use as nurseries in such situations remains to be determined.

**Acanthuridae.***Acanthuruschirurgus* (Bloch, 1787) has been considered a nursery species that uses other nursery habitats as well as mangroves and seagrasses. This species occurs on all the reefs we considered here, although it is not as common as the other two members of its genus, which are not considered to be nursery species. There is no clear evidence that availability of mangrove or seagrass has any strong influence on its abundance across different reef systems.

**Sphyraenidae.***Sphyraenabarracuda.* This pelagic species is thought to be mangrove-nursery dependent but also uses seagrass beds (Aiken et al. 2015). It ranges widely across expansive continental shelves, such as that on the west side of Florida, and travels hundreds of kilometers in the open ocean (Hansen and Kerstetter 2015), where it is taken as bycatch by tuna purse-seiners (e.g., Torres-Irineo et al. 2014). Seagrass beds acting as nurseries could support populations on reefs that lack mangroves, including Alacranes reef (see Hildebrand et al. 1964). However, adult dispersal of this large, mobile pelagic fish can account for its occurrence at all isolated locations that lack both nursery habitats, including submerged banks and reefs isolated by deep water from shallow areas containing such habitats (Tables 3–5).

**Conclusions about reliance on mangroves and seagrasses as nursery habitats**. Even though abundances of the 16 nursery species vary on reefs that have both nursery habitats adjacent to them, patterns of occurrence at isolated shallow reefs that lack mangroves and, sometimes, seagrasses indicate that distributions of only three of 16 nursery species of reef fishes on different reefs are consistent with their being highly dependent on such habitats. Those three are *Haemulonparra*, *H.sciurus*, and *Lutjanusgriseus*, which appear to be dependent on seagrass beds as they are lacking on oceanic reefs without such habitat that are also isolated by deep water from immigration. *Lutjanusgriseus* evidently has the ability to migrate long distances from shoreline nurseries to isolated reefs on continental shelves. We recognize that the information on abundances of others of those species that we presented here is relatively crude, and preliminary. Whether the density of populations of the other 13 nursery-species is lower on reefs lacking those nursery habitats remains to be determined, through use of similar methods of quantification of their abundances across a range of reef types. Conclusions of a number of previous studies that have focused on the dependency of Caribbean nursery-species on mangroves or seagrasses are limited in a number of ways: (i) They sometimes have been too narrowly focused on mangroves, rather than including seagrasses and other potential nursery habitats (but see Scharer 2007, 2009, Scharer et al. 2016) and (ii) have not attempted to examine reef systems that lack one or both nursery habitats, particularly reefs that are sufficiently isolated by deep water that immigration to them is highly unlikely (but see Scharer 2007, 2009, Scharer et al. 2016). (iii) All those studies have relied exclusively on observations rather than also employing specimen collections to enable accurate identification of newly recruited fishes (cf. Hildebrand 1964) and quantification of their abundance in different potential nursery habitats. Some reefs have both mangrove and seagrass habitats, others have seagrasses but no mangroves, but none likely have mangroves but not seagrasses as conditions sheltered enough to allow mangrove development invariably also allow seagrass development. This limits our ability to separate the relative influence of each of those two nursery habitats. Finally, the role of macroalgal beds as nursery habitat for Caribbean reef fishes thought to be reliant on mangroves and seagrasses needs evaluation. Eggertsen et al. (2017) found much higher densities of juvenile reef fishes, including some of the “nursery species” discussed here, in macroalgal beds than in beds of Shoal grass adjacent to Brazilian reefs (see also Evans et al. 2014, Tano et al. 2017). On the west Campeche reefs a number of reef fishes commonly seen elsewhere in seagrass beds were found associated with macroalgae: *Xyrichtyssplendens*, *Sparisomaatomarium* (Poey 1861), *S.radians* (Valenciennes in Cuvier and Valenciennes 1840), as well as small juveniles of *Sphyraenabarracuda* and *Ocyuruschrysurus*. Macroalgal beds clearly have the potential to act as suitable habitat for juveniles and adults of a number of reef fishes that commonly use seagrass beds.

While the reefs examined in the present study indicate that none of the 16 nursery species have an obligatory or even strong reliance on mangroves as nursery habitat, and that only a few may be strongly reliant on seagrasses, this does not invalidate the conclusions of previous studies of the importance of those habitats at sites for which the mangrove-nursery hypothesis was developed: Curacao and Bonaire. Those two islands rise abruptly from deep water and have only a very narrow rim of steeply sloping coral reef around their edges, with no sheltered habitat other than in large, peripheral inlets that contain mangroves and seagrasses. It may well be that those inlets provide all or nearly all suitable sheltered nursery habitat for *S.guacamaia* and some of the other nursery species on those islands. However, isolated reefs such as Alacranes reef (and Glover’s reef) are very different as it comprises a large, ~ 300 km^2^ oval of reef and shallow lagoon that host large areas of seagrass and shallow rocky substrata, with only a few tiny sand cays (Bello et al. 2005, Purkis et al. 2015). Oceanic islands like Curacao and Bonaire, shallow atolls like Alacranes and Glovers, and large submerged banks like Saba Bank represent extremes in terms of the absolute and relative abundances of different types of nursery habitat, the usage of which by nursery species may, in most cases, simply reflect their availability.

## Supplementary materials

Supplementary material 1 Figures S1–S10 Data type: multimedia Explanation note: **Figure S1.** Satellite image of Cayo Arenas reef complex. The lighthouse is on Isla Arenas, the cay on the western reef. A composite of satellite images by Pix4D, with permission. Notes: C = white center of cloud patch obscuring part of reef. SB = emergent sand bank. **Figure S2**. Cayo Arenas Island. Abbreviations: P = permanent pond. M = mangrove patch. From a satellite image by Pix4D, with permission. **Figure S3.** Mangrove patch at Cayo Arenas. Southerly view of patch of small mangroves exposed at low tide on south side of saltwater pond shown in Figure S2. White seabirds resting on rocks around left side of the mangrove patch provide scale for the size of the mangrove bushes. Photograph Quetzalli Hernandez. **Figure S4.** Satellite view of Triágulo Oeste reef with lighthouse cay. A composite of satellite images by Pix4D, with permission. **Figure S5**. Satellite view of Triángulo Este reef complex. A composite of satellite images by Pix4D, with permission. **Figure S6**. Drone view a section of Banco Obispo Norte. **Figure S7a,b.** Two drone views of Banco Obispo Sur, including 30 m long Isla Mujeres at anchor. **Figure S8**. Drone view of Banco Pera, with the 30 m long Isla Mujeres at anchor. **Figure S9**. Satellite view of the Cayo Arcas reef complex. The lighthouse is on the largest cay. A composite of satellite images by Pix4D, with permission. **Figure S10.** Mangroves at Cayo Arcas. Young mangrove plants immediately inshore of the reef crest at Cayo Arcas in 2018. Photograph Nuno Simões. This dataset is made available under the Open Database License (http://opendatacommons.org/licenses/odbl/1.0/). The Open Database License (ODbL) is a license agreement intended to allow users to freely share, modify, and use this Dataset while maintaining this same freedom for others, provided that the original source and author(s) are credited.D. Ross Robertson, Omar Domínguez-Dominguez, Yareli Margarita López Aroyo, Rigoberto Moreno Mendoza, Nuno Simões

## References

[B1] Aguilar-Perera A Tuz-Sulub A (2009) Occurrence of the Mardi Gras wrasse, *Halichoeresburekae* (Teleostei: Labridae) in the Alacranes Reef, off northern Yucatán Peninsula. Zootaxa 2298: 64–68.

[B2] Aguilar-Perera A Quijano-Puerto L Hernández-Landa RC (2016) Lionfish invaded the mesophotic coral ecosystem of the Parque Nacional Arrecife Alacranes, southern Gulf of Mexico. Marine Biodiversity 47: 15–16. 10.1007/s12526-016-0536-8

[B3] Aguilar-Perera A Hernández-Landa RC (2017) The rainbow parrotfish (*Scarusguacamaia*) does not depend on mangroves as nursery habitats in the Parque Nacional Arrecife Alacranes, Southern Gulf of Mexico. Marine Biodiversity 47: 13–14. 10.1007/s12526-016-0491-4

[B4] Aiken KA Dooley J Marechal J Pina Amargos F Russell B Singh-Renton S (2015) *Sphyraenabarracuda* (errata version published in 2017). The IUCN Red List of Threatened Species 2015: e.T190399A115319634. 10.2305/IUCN.UK.2015-4.RLTS.T190399A15603115.en

[B5] Belanger CL Jablonski D Roy K Berke SK Krug AZ Valentine JW (2012) Global environmental predictors of benthic marine biogeographic structure. PNAS 109: 14046–14051. 10.1073/pnas.1212381109 22904189 PMC3435205

[B6] Bello PJ Rios LV Liceaga CMA Zetina MC Cervera CK Arceo BP Hernandez NH (2005) Incorporating spatial analysis of habitat into spiny lobster (*Panulirusargus*) stock assessment at Alacranes reef, Yucatán, Mexico. Fisheries Research 73: 37–47. 10.1016/j.fishres.2005.01.013

[B7] Brandl SJ Goatley CHR Bellwood DR Tornabene L (2018) The hidden half: ecology and evolution of cryptobenthic fishes on coral reefs. Biological Reviews 93: 1846–1873. 10.1111/brv.12423 29736999

[B8] Chávez H (1966) Peces colectados en el Arrecife T Triángulo Oeste y en Cayo Arenas, Sonda de Campeche, Mexico. Acta Zoologica Mexicana 8: 1–12.

[B9] Chávez E Tunnell Jr JW Withers K (2007) Veracruz shelf and Campeche Bank. In: Tunnell Jr JW Chaez EA Withers K (Eds) Coral reefs of the southern Gulf of Mexico.Texas A&M Press, College Station, Texas, 41–67.

[B10] Chávez E Beaver CRK (2007) Reef fish. In: Tunnell Jr JW Chavez EA Withers K (Eds) Coral reefs of the southern Gulf of Mexico.Texas A&M Press, College Station, Texas, 102–111.

[B11] Choat JH Feitosa C Ferreira CE Gaspar AL Padovani-Ferreira B Rocha LA (2012) *Scarusguacamaia*. The IUCN Red List of Threatened Species 2012: e.T19950A17627624. 10.2305/IUCN.UK.2012.RLTS.T19950A17627624.en

[B12] Claydon JAB Calosso MC De Leo GA Peachey RBJ (2015) Spatial and demographic consequences of nursery-dependence in reef fishes: an empirical and simulation study 2015. Marine Ecology Progress Series 525: 171–183. 10.3354/meps11245

[B13] Cocheret de la Morinière E Polluxa BJA Nagelkerken I van der Velde G (2002) Post-settlement Life Cycle Migration Patterns and Habitat Preference of Coral Reef Fish that use Seagrass and Mangrove Habitats as Nurseries. Estuarine Coastal & Shelf Science 55: 309–321. 10.1006/ecss.2001.0907

[B14] Collette BB Williams JT Thacker CE Smith ML (2003) Shore Fishes of Navassa Island, West Indies: a case study on the need for rotenone sampling in reef fish biodiversity studies. Aqua 6: 89–131.

[B15] del Morales-Flores LF Tello-Musi JL Reyes-Bonilla H Pérez-España H Martínez-Pérez JA Horta-Puga G Velazco-Mendoza LA Álvarez del Castillo-Cárdenas AA (2013) Systematic checklist and zoogeographic affinities of ichthyofauna from Sistema Arrecifal Veracruzano, Mexico. Revista Mexicana de Biodiversidad 84: 825–846. 10.7550/rmb.34912

[B16] Dorenbosch M van Riel MC Nagelkerken I van der Velde G (2004) The relationship of reef Fish densities to the proximity of mangrove and seagrass nurseries. Estuarine coastal & shelf Science 60: 37–48. 10.1016/j.ecss.2003.11.018

[B17] Dorenbosch M Grol MGG Nagelkerken I van der Velde G (2006) Seagrass beds and mangroves as nurseries for the threatened Indo-Pacific Humphead wrasse, *Cheilinusundulatus* and Caribbean Rainbow parrotfish, *Scarusguacamaia* Biological Conservation 129: 277–282. 10.1016/j.biocon.2005.10.032

[B18] Dorenbosch M Verberk WCEP Nagelkerken I van der Velde G (2007) Influence of habitat configuration on connectivity between fish assemblages of Caribbean seagrass beds, mangroves and coral reef. Marine Ecology Progress Series 334: 103–116. 10.3354/meps334103

[B19] Eggertsen L Ferreira CEL Fontoura L Kautsky N Gulstrom M (2017) Seaweed beds support more juvenile reef fish than seagrass beds in a south-western Atlantic tropical seascape. Estuarine, Coastal and Shelf Science 196: 97–108. 10.1016/j.ecss.2017.06.041

[B20] Evans RD Wilson SK Field SN Moore JAY (2014) Importance of macroalgal fields as coral reef fish nursery habitat in north-west Australia. Marine Biology 161: 599–607. 10.1007/s00227-013-2362-x

[B21] Flores JS (1992) Vegetacion de Las Islas de La Peninsula De Yucatán: Floristica y Etnobotanica. Etnoflora Yucatanense, Fascículo 4. Universidad Autónoma de Yucatán, Mérida, 100 pp.

[B22] Friedlander AM Monaco ME Clark R Pittman SJ Beets J Boulon R Callender R Christensen J Hile SD Kendall MS Miller J Rogers C Stamoulis K Wedding L Roberson K (2013) Fish Movement Patterns in Virgin Islands National Park, Virgin Islands Coral Reef National Monument and Adjacent Waters. NOAA Technical Memorandum NOS NCCOS 172. Silver Spring, MD, 102 pp.

[B23] Garduño M (1988) Distribución de la Ictiofauna ascociada a los Arrecifes del Caribe Mexicano. MSc thesis, Centro de Investigaciones y de Estudios Avanzados el IPN. Unidad Merida, Yúcatan, México.

[B24] Garduño M Chávez EA (2000) Fish resource allocation in coral reefs of Yucatán Peninsula. Aquatic Ecosystems of Mexico: Status and Scope. In: Munawar M Lawrence SG Munawar IF Malley DF (Eds) Ecovision World Monograph Series 2000.Backhuys, Leiden, 367–381.

[B25] Goatley CHR González-Cabello A Bellwood DR (2016) Reef-scale partitioning of cryptobenthic fish assemblages across the Great Barrier Reef, Australia. Marine Ecology Progress Series 544: 271–280. 10.3354/meps11614

[B26] González-Gándara C (2014) Peces de Arrecife Blake, Veracruz, Mexico: Inventario, distribución y afinidades zoogeográficas. Ecosistemas y Recursos Agropecuarios 2: 87–97.

[B27] González-Gándara C Arias-González JE (2001) Lista actualizada de los peces del arrecife Alacranes, Yucatán, México. Anales del Instituto de Biología, Serie Zoología, Universidad Nacional Autonóma de México 72: 245–258.

[B28] González-Gándara C de la Cruz Francisco V Salas Pérez Dominguez Barradas C (2012) Lista de los peces de Tuxpan, Veracruz, México. Revista Científica UDO Agrícola 12: 675–689.

[B29] González-Gándara C Lozano Vilano M de L de la Cruz Francisco V Dominguez Barradas C (2013) Peces del sistema arrecifal Lobos-Tuxpan, Veracruz, Mexico. Universidad y Ciencia, Trópico Húmido 28: 191–208.

[B30] Green EP Short FT (2003) World Atlas of Seagrasses. University of California Press, Berkeley, 298 pp.

[B31] Halpern BS (2004) Are mangroves a limiting resource for two coral reef fishes. Marine Ecology Progress Series 272: 93–98. 10.3354/meps272093

[B32] Hansen NR Kerstetter DW (2015) Habitat use and vertical distribution of the great barracuda*Sphyraenabarracuda* (Edwards 1771) in the western north Atlantic using electronic archival tags. Gulf and Caribbean Research 26: SC4–SC9. 10.18785/gcr.2601.06

[B33] Harborne AR Nagelkerken I Wolff NH Bozec YM Dorenbosch M Grol MGG Mumby PJ (2015) Direct and indirect effects of nursery habitats on coral-reef fish assemblages, grazing pressure, and benthic dynamics. Oikos 125: 957−967. 10.1111/oik.02602

[B34] Hedgepeth JW (1954) Bottom communities of the Gulf of Mexico, U.S. Fishery Bulletin 89: 203–214.

[B35] Hernández-Landa RC Aguilar-Perera A (2018) Structure and composition of surgeonfish (Acanthuridae) and parrotfish (Labridae: Scarinae) assemblages in the south of the Parque Nacional Arrecife Alacranes, southern Gulf of Mexico. Marine Biodiversity 49: 647–662. 10.1007/s12526-017-0841-x

[B36] Hildebrand HH Chávez H Compton H (1964) Aporte al conocimiento de los Peces del Arrecife Alacranes, Yucatán (México). Ciencia (Mexico City) 23: 107–134.

[B37] Hitt S Pittman SJ Nemeth RS (2011) Diel movements of fishes linked to benthic seascape structure in a Caribbean coral reef ecosystem Marine Ecology Progress Series 427: 275–291. 10.3354/meps09139

[B38] Jones DL Walter JF Brooks EN Serafy JE (2010) Connectivity through ontogeny: fish population linkages among mangroves and coral reef habitats. Marine Ecology Progress Series 401: 245–258. 10.3354/meps08404

[B39] Lindeman K Anderson W Carpenter KE Claro R Cowan J Padovani-Ferreira B Rocha LA Sedberry G Zapp-Sluis M (2016a) *Lutjanusanalis*. The IUCN Red List of Threatened Species 2016: e.T12416A506350. 10.2305/IUCN.UK.2016-1.RLTS.T12416A506350.en

[B40] Lindeman K Anderson W Carpenter KE Claro R Cowan J Padovani-Ferreira B Rocha LA Sedberry G Zapp-Sluis M (2016b) *Lutjanusapodus*. The IUCN Red List of Threatened Species 2016: e.T155152A726254. 10.2305/IUCN.UK.2016-1.RLTS.T155152A726254.en

[B41] Lindeman K Anderson W Carpenter KE Claro R Cowan J Padovani-Ferreira B Rocha LA Sedberry G Zapp-Sluis M (2016c) *Lutjanusgriseus*. The IUCN Red List of Threatened Species 2016: e.T192941A2180367. 10.2305/IUCN.UK.2016-1.RLTS.T192941A2180367.en

[B42] Lindeman K Anderson W Carpenter KE Claro R Cowan J Padovani-Ferreira B Rocha LA Sedberry G Zapp-Sluis M (2016d) *Ocyuruschrysurus*. The IUCN Red List of Threatened Species 2016: e.T194341A2316114. 10.2305/IUCN.UK.2016-1.RLTS.T194341A2316114.en

[B43] Lindeman K Anderson W Carpenter KE Claro R Padovani-Ferreira B Rocha LA Sedberry G (2016e) *Haemulonflavolineatum*. The IUCN Red List of Threatened Species 2016: e.T194418A2333815. 10.2305/IUCN.UK.2016-1.RLTS.T194418A2333815.en

[B44] Littler DS Littler MM (2000) Caribbean Reef Plants. An identification guide to the reef plants of the Caribbean, Bahamas, Florida and Gulf of Mexico. Offshore Graphics. Washington DC, 542 pp.

[B45] Logan BW Harding JL Ahr WM Williams JD Snead RG (1969) Carbonate sediments and reefs, Yucatán shelf, Mexico. American Association of Petroleum Geologists Memoir 11: 1–198.

[B46] Lorda JF Athíe G V Camacho I Daessle LW Molina O (2019) The relationship between zooplankton distribution and hydrography in oceanic waters of the Southern Gulf of Mexico. Journal of Marine Systems. 192: 28–41. 10.1016/j.jmarsys.2018.12.009

[B47] Machemer EGP Walter JF III Serafy JE Kerstetter DW (2012) Importance of mangrove shorelines for rainbow parrotfish *Scarusguacamaia*: habitat suitability modeling in a subtropical bay. Aquatic Biology 15: 87–98. 10.3354/ab00412

[B48] McClellan DB Miller GM (2002) Reef fish abundance, biomass, species composition, and habitat characterization of Navassa Island. In: Miller MW (Ed.) Status of reef resources of Navassa Island: cruise report Nov 2002. NOAA Technical Memorandum NMFS-SEFSC-501, 119 pp.

[B49] Miller MW (2002) Status of reef resources of Navassa Island: cruise report Nov 2002. NOAA Technical Memorandum NMFS-SEFSC-501, 24–42.

[B50] Mumby PJ Edwards AJ Arias-González JE Lindeman KC Blackwell PG Gall A Gorczynska MI Harborne AR Pescod CL Renken H Wabnitz CCC Llewellyn G (2004) Mangroves enhance the biomass of coral reef fish communities in the Caribbean. Nature 427: 533−536. 10.1038/nature02286 14765193

[B51] Muñoz RC Buckel CA Whitfield PE Viehman S Clark R Taylor JC Degan BP Hickerson EL (2017) Conventional and technical diving surveys reveal elevated biomass and differing fish community composition from shallow and upper mesophotic zones of a remote United States coral reef. PLoS ONE 12(11): e0188598. 10.1371/journal.pone.0188598 PMC5697833 29161314

[B52] Nagelkerken I (2009) Evaluation of nursery function of mangroves and seagrass beds for tropical decapods and reef fishes: patterns and underlying mechanisms. In: Nagelkerken I (Ed.) Ecological Connectivity among tropical coastal ecosystems.Springer, Netherlands, 357–399. 10.1007/978-90-481-2406-0_10

[B53] Nagelkerken I Dorenbosch M Verberk WCEP Cocheret de la Morinière E van der Velde G (2000a) Importance of shallow-water biotopes of a Caribbean bay for juvenile coral reef fishes: patterns in biotope association, community structure and spatial distribution. Marine Ecology Progress Series 202: 175–192. 10.3354/meps202175

[B54] Nagelkerken I van der Velde G Gorissen MW Meijer GJ van‘t Hof T den Hartog C (2000b) Importance of mangroves, seagrass beds and the shallow coral reef as a nursery for important coral reef fishes, using a visual census technique. Estuarine, Coastal and Shelf Science 51: 31–44. 10.1006/ecss.2000.0617

[B55] Nagelkerken I van der Velde G (2003) Connectivity between coastal habitats of two oceanic Caribbean islands as inferred by ontogentic shifts by coral reef fishes. Gulf and Caribbean Research 14: 43–59. 10.18785/gcr.1402.04

[B56] Nagelkerken I Kleijnen S Klop T van den Brand RACJ Cocheret de la Morniere E van der Velde G (2001) Dependence of Caribbean reef fishes on mangroves and seagrass beds as nursery habitats: a comparison of fish faunas between bays with and without mangroves/seagrass beds. Marine Ecology Progress Series 214: 225–235. 10.3354/meps214225

[B57] Nagelkerken I Huebert KB Serafy JE Grol MGG Dorenbosch M Bradshaw JA (2017) Highly localized replenishment of coral reef fish populations near nursery habitats. Marine Ecology Progress Series 568: 137–150. 10.3354/meps12062

[B58] Purkis S Casini G Hunt D Colpaert A (2015) Morphometric patterns in Modern carbonate platforms can be applied to the ancient rock record: Similarities between Modern Alacranes Reef and Upper Palaeozoic platforms of the Barents Sea. Sedimentary Geology 321: 49–69. 10.1016/j.sedgeo.2015.03.001

[B59] Robertson DR Allen GR (1981) *Stegastesmellis* (Emery et Burgess, 1974), le juvenile de la Demoiselle Caraïbe *Stegastesdiencaeus* (Jordan et Rutter, 1898). Revue Francaise Aquariologie 7: 109–112.

[B60] Robertson DR Cramer KL (2014) Defining and Dividing the Greater Caribbean: Insights from the Biogeography of Shorefishes. Plos ONE 9: 1–16. 10.1371/journal.pone.0102918 PMC4108436 25054225

[B61] Robertson DR Perez-España H Nuñez Lara E Puc Itza F Simões N (2016a) The fishes of Cayo Arcas (Campeche Bank, Gulf of Mexico): an updated checklist. ZooKeys 640: 139–155. 10.3897/zookeys.640.10862 PMC5240370 28138290

[B62] Robertson DR Simões N Gutiérrez Rodríguez C Piñeros VJ Perez-España H (2016b) An Indo-Pacific damselfish widely established in the southwest Gulf of Mexico: prospects for a wider, adverse invasion. Journal of the Ocean Science Foundation 19: 1–17.

[B63] Robertson DR Smith-Vaniz WF (2008) Rotenone: an essential but demonized tool for assessing marine fish diversity. Bioscience 58: 165–170. 10.1641/B580211

[B64] Robertson DR Smith-Vaniz WF (2010) Use of clove oil in collecting coral reef fishes for research. Marine Ecology Progress Series 401: 295–302. 10.3354/meps08374

[B65] Rocha LA Choat JH Clements KD Russell B Myers R Lazuardi ME Muljadi A Pardede S Rahardjo P (2012) *Scaruscoeruleus*. The IUCN Red List of Threatened Species 2012: e.T190709A17797173. 10.2305/IUCN.UK.2012.RLTS.T190709A17797173.en

[B66] Rocha LA Collette BB Grubbs D Pezold F Simons J Caruso J Carlson J McEachran JD Brenner J Tornabene L Chakrabarty P Robertson DR Claro R Carpenter KE Vega-Cendejas M Camarena-Luhrs T Espinosa-Perez H Jelks H Williams J Craig MT (2015) *Halichoeresburekae*. The IUCN Red List of Threatened Species 2015: e.T187608A1826968. 10.2305/IUCN.UK.2015-4.RLTS.T187608A1826968.en

[B67] Rocha LA Choat JH Clements KD Russell B Myers R Lazuardi ME Muljadi A Pardede S Rahardjo P (2012) *Scarusiseri*. The IUCN Red List of Threatened Species 2012: e.T190732A17782171. 10.2305/IUCN.UK.2012.RLTS.T190732A17782171.en

[B68] Sandin S (2002) Reef fish trophic analysis from Navassa Island: Exploring biotic and anthropogenic factors In: Miller MW (Ed.) Status of reef resources of Navassa Island: cruise report Nov 2002.NOAA Technical Memorandum NMFS-SEFSC-501, 43–56.

[B69] Scharer MT (2006) Mona Island Reef Fish Community Structure and Function for Marine Protected e Area (MPA) Design Distribution of schooling snappers and grunts. Second Annual Symposium for Coastal and Marine Applied Research University of Puerto Rico Sea Grant College Program October 5, 2006, Mayagüez, Puerto Rico, 1–24. https://seagrantpr.org/wp-content/uploads/2014/11/Scharer.pdf

[B70] Scharer MT (2009) Using landscape ecology to describe habitat connectivity for coral reef fishes. PhD thesis, University of Puerto Rico, Mayagüez, 202 pp.

[B71] Scharer MT Nemeth MI Appeldoorn RS (2007) Mapping Ontogenetic Habitat Shifts of Coral Reef Fish at Mona Island, Puerto Rico. Proceedings of the 60^th^ Gulf and Caribbean Fisheries Institute November 5–9, 2007 Punta Cana, Dominican Republic: 305–310.

[B72] Serafy JE Faunce CH Lorenz JJ (2003) Mangrove shoreline fishes of Biscayne Bay, Florida. Bulletin of Marine Science 72: 161–180.

[B73] Serafy JE Shideler GS Araújo RJ Nagelkerken I (2015) Mangroves enhance reef fish abundance at the Caribbean regional scale. PLoS ONE 10: e0142022. 10.1371/journal.pone.0142022 PMC4633132 26536478

[B74] Short FT Carruthers TJR van Tussenbroek B Zieman J (2010a) *Thalassiatestudinum*. The IUCN Red List of Threatened Species 2010: e.T173346A6995927. 10.2305/IUCN.UK.2010-3.RLTS.T173346A6995927.en

[B75] Short FT Carruthers TJR van Tussenbroek B Zieman J (2010b) *Syringodiumfiliforme*. The IUCN Red List of Threatened Species 2010: e.T173378A7003203. 10.2305/IUCN.UK.2010-3.RLTS.T173378A7003203.en

[B76] Short FT Carruthers TJR van Tussenbroek B Zieman J (2010c) *Halodulewrightii*. The IUCN Red List of Threatened Species 2010: e.T173372A7001725. 10.2305/IUCN.UK.2010-3.RLTS.T173372A7001725.en

[B77] Smith-Vaniz WF Jelks HL Rocha LA (2006) Relevance of cryptic fishes in biodiversity assessments: A case study at Buck Island Reef National Monument, St. Croix. Bulletin of Marine Science 79: 17–48.

[B78] Strindberg S Coleman RA Burns Perez VR Campbell CL Majil I Gibson J (2016) In-water assessments of sea turtles at Glovers Reef Atoll, Belize. Marine Ecology Progress Series 31: 211–225. 10.3354/esr00765

[B79] Tano SA Eggertsen M Wikstrom SA Berkstrom C Buriyo AS Halling C (2017) Tropical seaweed beds as important habitats for juvenile fish. Marine and Freshwater Research 68: 1921–1934. 10.1071/MF16153

[B80] Torres-Irineo E Amande MJ Gaertner D Delgado de Molina A Murua H Chavance P Ariz J Ruiz J Lezama-Ochoa N (2014) Bycatch species composition over time by tuna purseseine fishery in the eastern tropical Atlantic Ocean Biodiversity and Conservation 23: 1157–1173. 10.1007/s10531-014-0655-0

[B81] Toller W Debrot AO Vermeij MJA Hoetjes PC (2010) Reef Fishes of Saba Bank, Netherlands Antilles: Assemblage Structure across a Gradient of Habitat Types. PLoS ONE 5(5): 1–13. e9207. 10.1371/journal.pone.0009207 PMC2873942 20502637

[B82] Tunnell Jr JW (2007) Island Biota. In: Tunnell Jr JW Chávez EA Withers K (Eds) Coral reefs of the southern Gulf of Mexico.Texas A&M Press, College Station, Texas, 119–125.

[B83] Tunnell Jr JW Chávez EA Withers K (2007) Coral reefs of the southern Gulf of Mexico. Texas A&M Press, College Station, Texas, 194 pp.

[B84] Tunnell Jr JW Chapman BR (2001) Seabirds of the Campeche Bank Islands, southeastern Gulf of Mexico. Atoll Research Bulletin 482: 1–50.

[B85] Verweij MC Nagelkerken I Hans I Ruseler SM Mason PRD (2008) Seagrass nurseries contribute to coral reef fish populations. Limnology & Oceanography 53: 1540−1547. 10.4319/lo.2008.53.4.1540

[B86] Weaver DC Rocha LA (2007) A new species of *Halichoeres* (Teleostei: Labridae) from the western Gulf of Mexico. Copeia 2007: 798–807. 10.1643/0045-8511(2007)7[798:ANSOHT]2.0.CO;2

[B87] Williams JT Carpenter KE Van Tassell JL Hoetjes P Toller W Etnoyer P Smith M (2010) Biodiversity Assessment of the Fishes of Saba Bank Atoll, Netherlands Antilles. PLoS ONE 5(5): 1–37. e10676. 10.1371/journal.pone.0010676 PMC2873961 20505760

[B88] Zarco-Perelló S Moreno Mendoza R Simóes N (2014) Checklist of fishes from Madagascar Reef, Campeche Bank, México. Biodiversity Data Journal 2: e1100. 10.3897/BDJ.2.e1100 PMC4040402 24891834

